# FGF7 enhances the expression of ACE2 in human islet organoids aggravating SARS-CoV-2 infection

**DOI:** 10.1038/s41392-024-01790-8

**Published:** 2024-04-23

**Authors:** Hao Meng, Zhiying Liao, Yanting Ji, Dong Wang, Yang Han, Chaolin Huang, Xujuan Hu, Jingyi Chen, Hengrui Zhang, Zonghong Li, Changliang Wang, Hui Sun, Jiaqi Sun, Lihua Chen, Jiaxiang Yin, Jincun Zhao, Tao Xu, Huisheng Liu

**Affiliations:** 1 School of Biomedical Engineering, Guangzhou Medical University, Guangzhou, 511495 Guangdong China; 2 Guangzhou National Laboratory, Guangzhou, 510320 Guangdong China; 3 State Key Laboratory of Respiratory Disease, National Clinical Research Center for Respiratory Disease, Guangzhou Institute of Respiratory Health, The First Affiliated Hospital of Guangzhou Medical University, Guangzhou, 510120 China; 4 Center for Translational Medicine, Wuhan Jinyintan Hospital, Wuhan, 430023 Hubei China; 5 School of Biomedical Sciences and Engineering, South China University of Technology, Guangzhou, 510006 Guangdong China

**Keywords:** Infection, Embryonic stem cells, Infectious diseases

## Abstract

The angiotensin-converting enzyme 2 (ACE2) is a primary cell surface viral binding receptor for SARS-CoV-2, so finding new regulatory molecules to modulate ACE2 expression levels is a promising strategy against COVID-19. In the current study, we utilized islet organoids derived from human embryonic stem cells (hESCs), animal models and COVID-19 patients to discover that fibroblast growth factor 7 (FGF7) enhances ACE2 expression within the islets, facilitating SARS-CoV-2 infection and resulting in impaired insulin secretion. Using hESC-derived islet organoids, we demonstrated that FGF7 interacts with FGF receptor 2 (FGFR2) and FGFR1 to upregulate ACE2 expression predominantly in β cells. This upregulation increases both insulin secretion and susceptibility of β cells to SARS-CoV-2 infection. Inhibiting FGFR counteracts the FGF7-induced ACE2 upregulation, subsequently reducing viral infection and replication in the islets. Furthermore, retrospective clinical data revealed that diabetic patients with severe COVID-19 symptoms exhibited elevated serum FGF7 levels compared to those with mild symptoms. Finally, animal experiments indicated that SARS-CoV-2 infection increased pancreatic FGF7 levels, resulting in a reduction of insulin concentrations in situ. Taken together, our research offers a potential regulatory strategy for ACE2 by controlling FGF7, thereby protecting islets from SARS-CoV-2 infection and preventing the progression of diabetes in the context of COVID-19.

## Introduction

The management of COVID-19 is still a challenge due to the SARS-CoV-2 variants continue to emerge.^1^ No matter how the variants mutation happened in the virus, the interaction between the spike protein of SARS-CoV-2 and the primary host cell membrane receptor, angiotensin-converting enzyme 2 (ACE2) is indispensable for viral attachment and entry.^2,3^ Accordingly, tissues with higher expression of ACE2 are more susceptible to COVID-19 infection than tissues with lower expression.^4,5^ Hence, reducing ACE2 expression is a robust strategy for impeding SARS-CoV-2 binding to host cells. Several small molecules and FDA-approved drugs, such as short-chain fatty acids (SCFAs), ursodeoxycholic bile acid (UDCA), UDCA combined with dihydroartemisinin (DHA), corticosteroids and fluticasone propionate, have been reported to prevent SARS-CoV-2 infection through the reduction of ACE2 expression in various cells, tissues and organs.^6–8^ Therefore, identifying new regulatory factors that modulate ACE2 expression holds great promise for the development of drugs and vaccines for the treatment of COVID-19.

Fibroblast growth factors (FGFs) control cellular proliferation, inflammation, angiogenesis, tissue repair, and regeneration by interacting with FGF receptors (FGFR1, 2, 3 and 4).^9^ FGF production is tightly regulated under homeostatic conditions via its sequestration in the extracellular matrix (ECM) reservoir.^10^ However, in the context of COVID-19, proteases liberate ECM-bound FGFs to participate in the pathophysiological process associated with inflammation and angiogenesis-dependent diseases.^11,12^ Recent studies have indicated that FGFs aggravate SARS-CoV-2 infection in host cells;^13–16^ Inhibiting of FGFRs with dobesilate could improve the opacity of the hemithorax without the need for oxygen therapy in COVID-19 patients.^12^ Additionally, a correlation between FGFs and ACE2 has been reported. For example, FGF21 and FGF23 have been shown to regulate ACE2 enzymatic activity and levels in a rodent model.^17–20^ This correlation between FGFs and ACE2 suggests that FGFs are potential regulatory factors that modulate ACE2 levels. FGFs are distributed heterogeneously in human tissues, among them FGF7 plays a crucial role in pancreatic development, regeneration and the survival of implanted islets.^21^ Taken together, in COVID-19 patients, especially those with comorbid diabetes, the potential regulatory influence of FGFs on ACE2 expression in pancreatic islets and the role of this process in islet function and viral infection has not been explored.

Increasing evidence and meta-analyses of COVID-19 progress indicate that the acute phase of respiratory symptoms, a significantly larger number of individuals with COVID-19 develop new-onset diabetes compared to uninfected individuals.^22^ Further evidences have shown that SARS-CoV-2 directly infects pancreatic β-cells through ACE2, impairing their insulin secretion capability.^23–26^ At the onset of COVID-19 pandemic, there were concerns regarding anti-diabetic drugs such as angiotensin II receptor blockers (ARBs) and ACE inhibitors, which increase the expression and activity of ACE2, potentially raising the risk for diabetic patients.^27^ However, retrospective clinical studies have not found evidence that these antidiabetic drugs worsen COVID-19 prognosis.^28,29^ In addition to functioning as viral binding receptors, ACE2 also improves insulin secretion and has anti-oxidant and anti-inflammatory effects.^30,31^ Most information about the regulatory effect of ACE2-Ang (1–7) on β cell function and insulin secretion has been obtained from animal models^32^ and β cell lines.^30,31^ Overall, a comprehensive understanding of the changes in ACE2 levels within human islets, particularly in β cells, and the resultant effects on β cell function and viral infection during SARS-CoV-2 infection is lacking.

Based on the above proposal, we utilize hESC-derived islet organoids, animal models and COVID-19 patients to elucidate the effect of FGF7 on ACE2 levels within the islets, and its implications for insulin secretion and SARS-CoV-2 infection.

## Results

### Dynamic expression of ACE2 during islet development

**Fig. 1:**
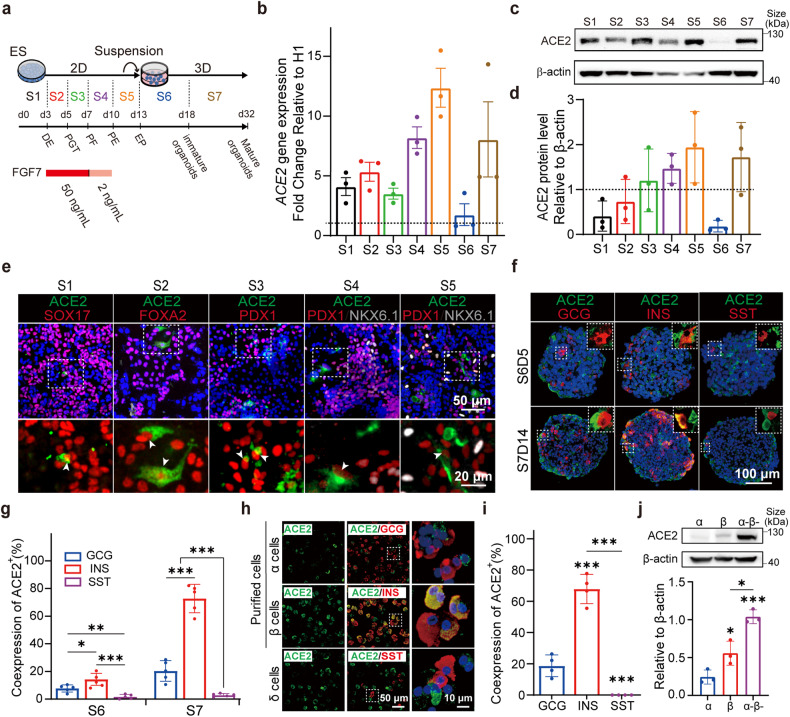
Dynamic expression of ACE2 during the differentiation of human islet organoids from hESCs. **a** Schematic diagram outlining the differentiation protocol. The cells from the ES to S5 stages were subjected to 2D culture and the cells in S6 and S7 were suspended and 3D aggregated at the first day of S6. ES embryonic stem cells, DE definitive endoderm, PGT primitive gut tube, PF posterior foregut, PE pancreatic endocrine, EP endocrine progenitor. **b** Dynamic changes in *ACE2* mRNA expression relative to ES from S1 to S7 relative to that in the ES stage. The dashed line indicates *ACE2* mRNA expression in ES cells, which was set as 1. **c** Western blot analysis of ACE2 protein levels relative to that of β-actin from S1 to S7. **d** Quantification of the ACE2 protein level relative to that of β-actin from S1 to S7. The dashed line represents the intensity of β-actin, which was set to 1. **e** Immunofluorescence (IF) staining of differentiated cells from each stage for ACE2 (green) and stage-specific markers (red or white, S1: SOX17; S2: FOXA2; S3: PDX1; S4: PDX1 and NKX6.1; S5: PDX1 and NKX6.1). The white arrowheads represent the colocalization of ACE2 with stage-specific markers. The image at the bottom provides an enlarged perspective of the enclosed region within the dashed box. **f** Cryosections of islet organoids at S6D5 and S7D14 were subjected to IF staining for ACE2 and endocrine markers (GCG, INS, and SST). The image in the top-right corner of the picture represents an enlarged view of the region enclosed within the dashed box. **g** Quantification of (**f**) showing the percentage (%) of ACE2^+^ cells among GCG^+^, INS^+^, and SST^+^ cells in islet organoids. **h** IF staining of ACE2 in purified α (GCG^+^), purified β (INS^+^), and δ (SST^+^) cells from S7D14 islet organoids. The image on the right-hand side represents an enlarged view of the region enclosed within the dashed box. **i** Quantification of (**h**) revealing the percentages of ACE2^+^ cells among GCG^+^ cells, INS^+^ cells and SST^+^ cells. **j** Western blot image and quantification of ACE2 protein levels relative to β-actin in purified α cells, purified β cells and α^−^β^−^ cells (the remaining cells after sequentially sorting α and β cells) from S7D14 islet organoids. Images are from one representative experiment from 3 to 5 independent experiments. For all statistical plots, the data are presented as mean ± SD, the distinct dots are represented as the individual values of 3 to 5 replicates with 3 repeats in each experiment. *p* values were calculated by one-way ANOVA and Tukey’s multiple comparison test. *p < 0.05, **p < 0.01, ***p < 0.001

FGF7 was added at stage 2 (S2, 50 ng/ml), stage 3 (S3, 50 ng/ml) and stage 4 (S4, 2 ng/ml) of islet development according to several published islet organoid differentiation protocols^33,34^ (Fig. 1a). Precisely modulating the concentration and duration of FGF7 stimulation is critical for pancreatic lineage development and mimics in vivo fetal pancreatic development. Although FGF7 is important for pancreas development, whether FGF7 can induce and modulate ACE2 expression during islet differentiation has not been determined. Therefore, we first analyzed the dynamic trajectory of ACE2 expression during islet organoid differentiation from hESCs.

Gene (Fig. 1b) and protein (Fig. 1c, d) expression of ACE2 emerged at stage 1 (S1) and increased with differentiation, reaching a maximum in stage 5 (S5, endocrine progenitors). Intriguingly, both the gene and protein expression of ACE2 decreased precipitously in stage 6 (S6, immature islet organoid), which was likely due to the suspension procedure concentrating endocrine cells while reducing exocrine and other unknown cells. The self-assembly process results in the loss of a substantial number of non-endocrine cells, suggesting that the non-endocrine cells express more ACE2 than do endocrine cells. During the maturation of islet organoids in stage 7 (S7), the expression of ACE2 increased significantly (Fig. 1b–d). Furthermore, removing FGF7 from S2 to S4 (Supplementary Fig. 1a) resulted in a relatively flat ACE2 expression curve (Supplementary Fig. 1b, red curve) compared to that of FGF7-treated cells in S4 and S5 (Supplementary Fig. 1b, green curve). FGFRs (FGFR1-4) are receptors activated by FGF ligands, and their dynamic expression during islet organoid differentiation was also assessed. Our results indicated that FGFR2 is the predominant FGF receptor in cells in S4, S5 and S7 (Supplementary Fig. 1c), which is consistent with the trajectory of ACE2 expression during islet organoid development (Supplementary Fig. 1b). In summary, removing FGF7 from the differentiation medium silenced the FGF7-FGFR pathway, resulting in the elimination of ACE2 expression in S4 and S5, suggesting a potential role for FGF7 in the expression of FGFR2 and ACE2.

ACE2-positive cells were very rare in the early stages [from definitive endocrine (DE), primitive gut tubing (PGT), and pancreatic foregut (PF) to pancreatic endocrine (PE)] and exhibited co-staining for SOX17 at the DE stage, FOXA2 at the PGT stage and PDX1 at the PE and PF stages (Fig. 1e). In the endocrine progenitors at S5, ACE2-positive cells exhibited stronger co-staining for PDX1 but not NKX6.1 (Fig. 1e, S5). Szlachcic et al. summarized ACE2 distribution at mRNA level in hESC-derived islet progenitors^35,36^ and fetal pancreas^37^ by analysis of the existed single-cell RNA sequencing (scRNA-seq) datasets. The results from different datasets all suggest low ACE2 mRNA expression, which is detectable only in a small subset of progenitors. Moreover, immunofluorescence (IF) staining revealed the presence of ACE2 on the cell membrane of PDX1^+^ early pancreatic progenitor cells.^24^ Hence, our findings combined with those of previous studies indicate the SARS-CoV-2 receptor ACE2 is expressed on human pancreatic progenitors and that its expression dynamically varies with the development of endocrine progenitors.

Previously published scRNA-seq datasets^38,39^ of the human pancreas revealed abundant expression of neuropilin 1 (*NRP1*), transferrin receptor (*TFRC*), and *FURIN*, but relatively lower expression of ACE2 and transmembrane serine protease (*TMPRSS2*) in the pancreas. In this study, we assessed the dynamic changes in the mRNA expression levels of SARS-CoV-2 related receptors (*NRP1* and *TFRC*) and proteases (*FURIN* and *TMPRSS2*) during islet development. Intriguingly, the gene expression of *ACE2* peaked at S5, whereas *NRP1*, *TFRC*, *FURIN* and *TMPRSS2* exhibited their highest gene expression in mature islet organoids (S7) (Supplementary Fig. 1d). Moreover, unlike the sharp decrease in *ACE2* expression at S6, the gene expression of the two receptors, *NRP1* and *TFRC*, did not decrease significantly. Additionally, the expression of the two proteases, *FURIN* and *TMPRSS2*, increased by fivefold and twofold, respectively, in S6 compared to S5. These results indicated that eliminating nonendocrine cells did not affect the gene expression of *NRP1*, *TFRC*, *FURIN* or *TMPRSS2*. The scRNA-seq data for endocrine progenitors (EP) cells differentiated from hPSCs demonstrated that TMPRSS2 and NRP1 were expressed in different types of progenitors.^24^

### ACE2 is predominantly expressed in β cells rather than in α and δ cells

To investigate the co-localization of ACE2 within hESC-derived islets (α, β, and δ cells), we performed co-staining for ACE2 and GCG, INS, and SST in islet organoids obtained at S6 and S7 (Fig. 1f). At S6, ACE2 was expressed in 7.7 ± 2.6% of GCG^+^ cells, 14 ± 4.3% of INS^+^ cells, and 1.8 ± 1.6% of SST^+^ cells. With the maturation of islet organoids in S7, the percentage of ACE2-positive cells increased to 20 ± 7.4% in GCG^+^ cells, 73 ± 10% in INS^+^ cells, and 2.9 ± 1.2% in SST^+^ cells, respectively (Fig. 1g, S7). These results revealed the co-localization of ACE2 was highest in β cells, moderate in α cells but negligible in δ cells, which was consistent with the previous findings on hESC-derived islet organoids.^40^ Our results indicated that ACE2 expression did not appear at the initial appearance of INS^+^ cells but rather that ACE2 expression occurred during the maturation of islet organoids. Therefore, the heterogeneous and dynamic expression pattern of ACE2 during pancreatic islet development suggests that it may be modulated by additional regulatory molecules.

To discern the ACE2 distribution patterns in the three major endocrine cell populations individually as single cells rather than intermingled in a 3D aggregate, we purified and enriched α and β cells using magnetic microbeads coated with CD26^41^ and CD49a^42^ antibodies, respectively. The mRNA and whole cell protein samples of purified α and β cells, and the remaining cells (α-β-) were collected for subsequent assays. Evaluation of sorting efficiency via IF staining indicated that more than 70% of the purified α and β cells were positively stained for GCG and INS, respectively (Supplementary Fig. 1e). qPCR analysis revealed that *GCG* expression was predominantly observed in purified α cells, with levels ~3 times greater than those in purified β cells, and 560 times greater than those in α-β-cells (Supplementary Fig. 1f, left). Conversely, the expression of *INS* exhibited 7.2-and 197-fold greater in purified β cells than in purified α cells and α-β- cells, respectively (Supplementary Fig. 1f, middle). Notably, δ cells that expressed the *SST* gene were not retained among the α-β- cells as supposed. Instead, *SST* was primarily expressed in purified β cells, at levels ~5 times and 165 times greater than those in α cells and α-β- cells, respectively (Supplementary Fig. 1f, right). Consistently, IF staining (Supplementary Fig. 1g) revealed the presence of a substantial percentage of δ cells (17%) in the purified β cells. Importantly, IF staining for INS and SST did not reveal co-localization of the corresponding cells.

Co-staining of ACE2 with hormonal cell markers (GCG, INS, and SST) confirmed a co-localization rate of ~75% with ACE2 in purified β cells; this percentage was relatively low in purified α cells (20%), and co-localization was absent in δ cells (Fig. 1h, i). Our findings suggested that ACE2 expression in CD49a^+^ cells were the predominantly contributor to ACE2 expressed in β cells, supported by the negligible ACE2 expression observed in δ cells. Western blot (WB) analysis of ACE2 in purified cells objectively revealed the total protein concentration of ACE2 was higher in purified β cells compared to α cells (Fig. 1j), corroborating the greater co-localization of ACE2 with β cells than with α cells (Fig. 1h). Moreover, gene expression analysis of FGFRs in purified α and β cells revealed that FGFR2 expression was greater in β cells than in α cells (Supplementary Fig. 1h). In summary, our study established the hESC-derived islet organoids as an ideal in vitro model system for studying ACE2 expression in pancreatic endocrine cells. This model recapitulates the heterogeneous expression and distribution of ACE2 observed in the in vitro islet development system, offering a reliable platform to investigate viral infection mechanisms and islet function. Moreover, our findings suggested a potential correlation between FGFR2 and ACE2 based on their similar expression patterns during islet development. Finally, the variable expression of ACE2 in the islet organoids suggested the feasibility of studying ACE2 regulation using this in vitro model.

Consequently, we investigated the gene expression profiles of *NRP1, TFRC, TMPRSS2*, and *FURIN* in purified α, β, and α-β- cells. Remarkably, both SARS-CoV-2 receptors, the *NRP1* and *TFRC*, exhibited similar gene expression levels in the purified α, β, and α-β- cells groups (Supplementary Fig. 1i). However, both SARS-CoV-2 proteases, the *TMPRSS2* and *FURIN*, indicated significantly greater expressions in both α and β cells than in α-β- cells. Taken together, our findings highlight ACE2 as the sole SARS-CoV-2 receptor with increased expression in β cells in accordance with the increased gene expression of FGFR2.

### FGF7 regulates ACE2 expression in pancreatic progenitors primarily through FRFR2 receptor

**Fig. 2:**
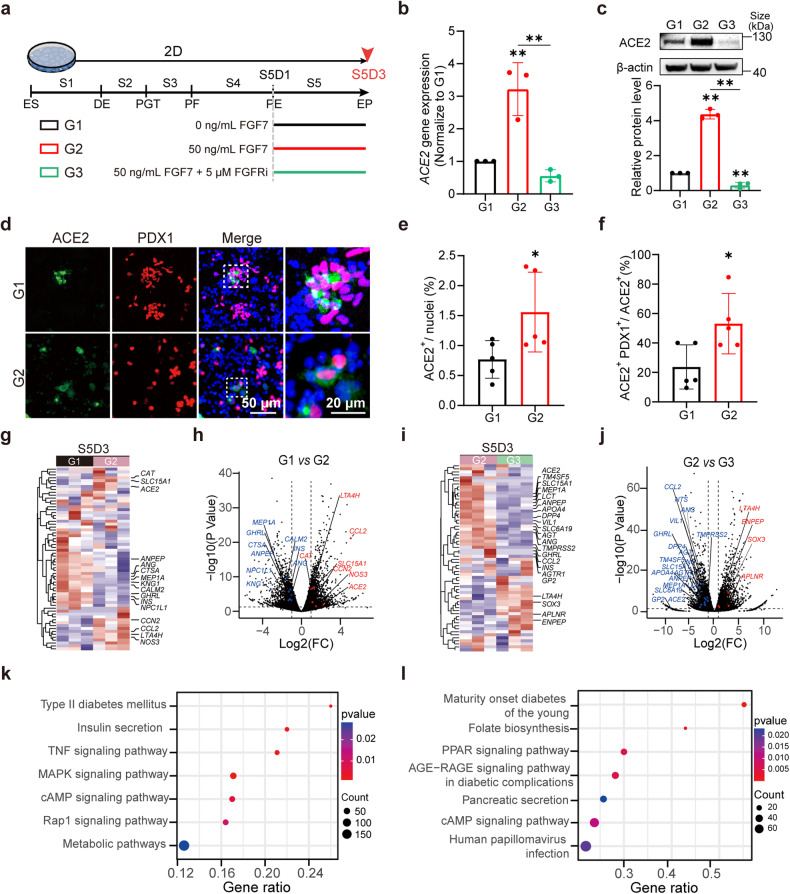
FGF7 regulates ACE2 expression during the early developmental stages of hESC-derived pancreatic endocrine progenitors via the FGF7-FGFR2/1 pathway. **a** Scheme of the dosages of FGF7 and FGFR inhibitors (FGFRis, including FGFR1 and FGFR2 inhibitors) administered to endocrine progenitors in group 1 (G1, control, without FGF7 and FGFRi), group 2 (G2, 50 ng/ml FGF7) and group 3 (G3, 50 ng/ml FGF7 + 5 μM FGFRis). The red arrow represents the sample collection time at S5D3. **b** *ACE2* mRNA expression in endocrine progenitors from the G1, G2 and G3 groups. **c** Western blot analysis of ACE2 protein levels in the G1, G2 and G3 groups. **d** Co-localization of ACE2 with the stage-specific marker PDX1 after FGF7-treatment in S5. Green, ACE2; red, PDX1; blue, DAPI. **e** Quantification of (**d**) showing the ratio of ACE2^+^ cells relative to total nuclei in the G1 and G2 groups. **f** Quantification of (**d**) showing the ratio of PDX1^+^ and ACE2^+^ double-positive cells (PDX1^+^ ACE2^+^) relative to ACE2^+^ cells in G1 and G2. **g** Heatmap illustrating the proximity of G1 to G2. Significant ACE2-related genes are labeled on the right. **h** Volcano plots showing selected genes in the comparison of G1 and G2. Significant ACE2-related genes with *p* < 0.05 and log2 (fold change) >1 are highlighted in red, while log2 (fold change) < −1 are highlighted in blue. **i** Heatmap illustrating the proximity of G2 to G3. Significant ACE2-related genes are labeled on the right. **j** Volcano plots showing selected genes in the comparison of G2 and G3. Significant ACE2-related genes with *p* < 0.05 and log2 (fold change) >1 are shown in red while those with log2 (fold change) < −1 are shown in blue. **k** Selection of significantly enriched KEGG gene sets in G1 versus G2. **l** Selection of significantly enriched KEGG gene sets in G2 versus G3. Images are from one representative experiment from 3 to 5 independent experiments. For all statistical plots, the data are presented as mean ± SD, the distinct dots are represented as the individual values of 3 to 5 replicates. *p* values were calculated using an unpaired Student’s *t* test (**e**, **f**) or one-way ANOVA and Tukey’s multiple comparison test (**b**, **c**). *p < 0.05, **p < 0.01, ***p < 0.001

Based on the parallel patterns observed in the trajectories of FGFR2 and ACE2 during pancreatic development, we postulated a potential correlation between FGF7-FGFR2 signaling and ACE2 expression. To investigate this possible correlation, FGF7 (Fig. 2a, G2 group) and its receptor FGFR inhibitors (FGFRis, PD166866 and alofanib; Fig. 2a, G3 group) were added to the culture medium of endocrine cells, while non-treated cells served as the control (G1). Our findings revealed a significant increase in both the gene and protein levels of ACE2 in endocrine progenitors upon exposure to FGF7 (Fig. 2b, c). To identify the specific cell types affected by FGF7-induced ACE2 upregulation, we co-stained ACE2 (in green) with the stage-specific marker PDX1 (in red) (Fig. 2d). Our analysis indicated a substantial increase in the number of ACE2^+^ cells compared to that in the non-treated control group (Fig. 2e, *p* < 0.05). Additionally, FGF7 treatment significantly increased the percentage of ACE2^+^ cells among PDX1^+^ cells from 20 to 55% (Fig. 2f, *p* < 0.05). In summary, our findings suggest that FGF7 is involved in the upregulation of ACE2 expression specifically within PDX1-positive endocrine progenitors.

There are 18 mammalian FGFs (FGF1-FGF10 and FGF16-FGF23) categorized into six families, along with 4 FGFRs (FGFR1–4)^43^ (Supplementary Fig. 2a), which exhibited heterogeneous expression across various cell types in vivo. These FGFs demonstrate diverse binding preferences for different FGFRs in each subfamily. Despite our focus on FGF7 in this study, it remains unclear whether other FGFs also regulate ACE2 expression or whether the observed modulatory effect is specific to FGF7. Hence, FGFs from different subfamilies were utilized to investigate their effects on ACE2 expression. Among them, FGF2, FGF4, and FGF7, which primarily interact with FGFR1c and FGFR2b, significantly upregulated ACE2 expression compared to that in the control (*p* < 0.01; Supplementary Fig. 2b). In contrast, FGF8 and FGF9, which interact primarily with FGFR3c, did not significantly upregulate ACE2. Additionally, FGF21 from the FGF19 subfamily failed to significantly upregulate ACE2, possibly due to its weaker activation of FGFRs. In summary, FGFs primarily activate ACE2 expression by interacting with FGFR2 and FGFR1. Among these FGFs, members of the FGF7 subfamily is the only ones that specifically interact with FGFR2 and FGFR1. To evaluate the efficacy of various types of FGFR1 and FGFR2 inhibitors in regulating ACE2 expression, we screened a series of FGFR1 inhibitors (e.g., PD, NSC, and SU) and FGFR2 inhibitors (SKLB and alofanib). Our results indicated that most FGFR1 and FGFR2 inhibitors partially counteracted FGF7-induced ACE2 overexpression (*p* < 0.05, Supplementary Fig. 2c). Notably, the combination of FGFR1 and FGFR2 inhibitors (alofanib: a specific FGFR2 allosteric agent; PD: a specific FGFR1 inhibitor) most significantly reversed the FGF7-induced overexpression of ACE2 (*p* < 0.01, Fig. 2b, c). In summary, FGF7 regulates ACE2 expression through FGFR1 and FGFR2, which can be efficiently counteracted by the combination of FGFR2 and FGFR1 inhibitors.

Notably, most FGFR inhibitors lack specificity and might interfere with other signaling pathways. To address this concern, we employed more specific siRNAs targeting FGFR1 and FGFR2 to silence the expression of these receptors. Our results demonstrated successful knockdown of *FGFR1* and *FGFR2*, with a 50% decrease in expression compared to that in the control (Supplementary Fig. 2d, e). Additionally, *ACE2* expression significantly decreased after treatment with siFGFR1 and siFGFR2 (*p* < 0.05 and *p* < 0.01, respectively) (Supplementary Fig. 2f). Subsequently, we added FGF7 to evaluate ACE2 levels after FGFR1 and FGFR2 were knocked down. Knockdown of FGFR1 alone did not block FGF7-induced ACE2 upregulation (Supplementary Fig. 2g). However, knocking down (KD) FGFR2, both individually and in combination with FGFR1 KD, significantly blocked FGF7-induced ACE2 upregulation. These results provide further evidence that FGFR2 plays a primary role in determining ACE2 expression in the endocrine progenitor cells. FGFR1 appears to exert a smaller effect on ACE2 expression, particularly in the presence of FGF7 treatment. These findings strongly indicate a direct correlation between FGFR2 and ACE2 expression.

To further investigate the role and potential signaling pathways underlying the regulatory effect of FGF7 on the expression of *ACE2*, we conducted bulk RNA sequencing (RNA-seq) of endocrine progenitors treated with FGF7 and control cells. The complete ACE2 network (encompassing 68 genes) was constructed by Wicik et al.^44^ In the FGF7-treated cells, bulk RNA-seq revealed the upregulation of 7 genes and the downregulation of 9 genes (Fig. 2g, h) compared to the levels observed in the control cells. Conversely, cells treated with FGFR inhibitors presented 19 downregulated and 4 upregulated genes (Fig. 2i, j) compared to the levels of cells treated with FGF7. Remarkably, only four genes, chemokine ligand 2 (*CCL2*), *ACE2*, solute carrier family 15 member 1 (*SLC15A1*) and glycoprotein 2 (*GP2*), met the criteria for being enhanced by FGF7 and inhibited by an FGFR inhibitor. KEGG pathway analysis revealed enrichment of pancreas-related functions associated with type II diabetes mellitus, insulin secretion and metabolic pathways (Fig. 2k). Additionally, inflammation and cytokine-related terms, such as the TNF signaling pathway, which plays a vital role in *ACE2* transcriptional changes, tended to be enriched. The downstream pathways of FGF7-FGFR2, such as the MAPK pathway, were enriched in the differentially expressed genes. Notably, after FGFR was blocked by inhibitors, pancreas dysfunction-related pathways, such as the age-rage-signaling pathway, which is involved in diabetic complications, maturity-onset diabetes of the young (MODY), and pancreatic secretion, were found to be enriched in differentially expressed genes after blockade of the FGF7-FGFR2 signaling pathway (Fig. 2l). These findings strongly suggest a pivotal role of the FGF7-FGFR2 pathway in both islet development and function, and inhibition of the FGF7-FGFR2 pathway may increase the risk of diabetes-related diseases. In summary, FGF7 interacts primarily with FGFR2 and to a lesser extent with FGFR1, consequently upregulating ACE2 expression in pancreatic cells. Nevertheless, the precise mechanisms underlying the downstream pathway connecting FGFR2 and ACE2 remain insufficiently understood and warrant further investigation in future studies.

### FGF7 modulates ACE2 expression in islet organoid, especially in β cells

**Fig. 3:**
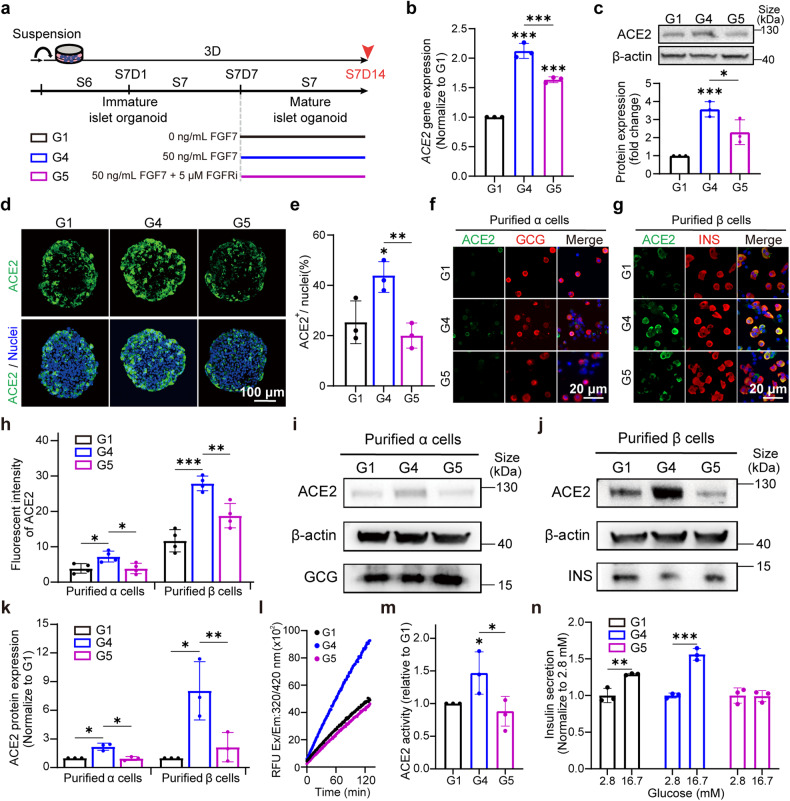
FGF7 regulates ACE2 expression and activity during the late stages of development in mature islet organoids, which affects β-cell function. **a** Scheme illustrating the dosage and duration of FGF7 and FGFRi treatment in Group 1 (G1, control), Group 4 (G4, treated with 50 ng/ml FGF7), and Group 5 (G5, treated with 50 ng/ml FGF7 + 5 μM FGFRi) in the late mature islet stage (S7). The red arrow represents the sample collection time at S7D14. **b** *ACE2* mRNA expression in mature islet organoids from G1, G4 and G5. **c** Western blot analysis of ACE2 protein levels in G1, G4 and G5. **d** IF staining of ACE2 (green) and nuclei (blue) in S7D14 islet organoids from G1, G4 and G5. **e** Quantification of (**d**) to calculate the number of ACE2^+^ cells in islet organoids divided by the numbers of nuclei. **f** Co-staining of ACE2 (green) and GCG (red) in purified α cells from G1, G4 and G5. **g** Co-staining of ACE2 (green) and INS (red) in purified β cells from G1, G4 and G5. **h** Measurement of the fluorescence intensity (FI) of (**f**) and (**g**) for ACE2^+^ cells in purified α and β cells (ACE2^+^/GCG^+^ or ACE2^+^/INS^+^ double-positive cells) from G1, G4 and G5. **i** Western blot images of ACE2, β-actin and GCG in purified α cells from G1, G4 and G5. **j** Western blot images of ACE2, β-actin and INS in purified β cells from G1, G4 and G5. **k** Quantification of (**i**) and (**j**) normalized to G1. **l** ACE2 enzymatic activity curves of islet organoids from G1, G4 and G5 subjected to a kinetics model for 130 min. **m** ACE2 enzymatic activity (mU) per mg protein relative to G1. **n** Insulin secretion of islet organoids in the static GSIS assay. Images are from one representative experiment from 3 to 4 independent experiments. The data are represented as the individual values of 3 to 4 replicates, with 3 replicates in each experiment. *p* values were calculated by one-way ANOVA and Tukey’s multiple comparison test. *p < 0.05, **p < 0.01, ***p < 0.001

FGFs regulate ACE2 expression through FGFR2 and FGFR1 in pancreatic progenitors, and this effect can be counteracted by FGFR1 and FGFR2 inhibitors. We established a novel ACE2 regulatory pathway in our pancreatic progenitors. However, whether FGF-FGFR2/1-ACE2 regulation is common in islet organoids and other types of cells/organs, and whether this function is limited to pancreatic progenitors remained to be investigated. To further examine the influence of FGF7 on ACE2 expression in mature islets (mimic adult islets), FGF7 and FGFR inhibitors were administered from S7D7 to S7D14 (Fig. 3a, G4 and G5 groups). Islet organoids at S7D7 were considered as fully developed functional mature islets.^34^ Treatment of islet organoids with FGF7 (G4) significantly increased in ACE2 expression at both the gene (2.5-fold, Fig. 3b) and protein (4-fold, Fig. 3c) levels. Similar to our observations in endocrine progenitors, the combination of FGFR1 and FGFR2 inhibitors effectively counteracted FGF7-induced ACE2 upregulation in the islet organoids. IF staining of cryosections of S7D14 islet organoids (Fig. 3d) demonstrated a significantly greater percentage of ACE2-positive cells (42.7%) in the G4 than in G1 (26.7%) (Fig. 3e). The addition of FGFR inhibitors significantly reduced the percentage of ACE2-positive cells in the islet organoids.

Purified α and β cells as opposed to islet-like organoids, eliminate the influence of miscellaneous cells, allowing for a more direct observation of the effects of FGF7 and FGFR inhibitors on ACE2 within these cells. IF co-staining of GCG, INS with ACE2 showed a substantial increase in the fluorescence intensity (FI) of ACE2 in isolated α and β cells following FGF7 treatment (Fig. 3f–h), and this increase was effectively attenuated by FGFR inhibitors. WB analysis revealed a significant upregulation of ACE2 by FGF7 (Fig. 3i–k, G4 group) in both purified α and β cells. Overall, ACE2 expression increased ~2-fold in purified α cells and 7-fold in purified β cells on average in G4 cells treated with FGF7 compared to that in G1 cells. FGFR inhibitors significantly reduce the expression of ACE2 in purified α and β cells from IF and WB results (Fig. 3f–k, G5 group). We have examined the gene expression of FGFRs in isolated α and β cells, both FGFR2 and FGFR1 are prominently expressed in these two endocrine cells (Supplementary Fig. 1h). The expression of FGFR1 is comparable, whereas FGFR2 expression in β cells is much higher than in α cells. Therefore, the higher level of FGFR2 is accompanied with higher level of ACE2 expression and be more sensitivity to FGF7 stimulation. In summary, FGF7 predominantly enhances ACE2 expression in β cells and moderately affects α cells. The addition of FGFR inhibitors more efficiently inhibit the ACE2 expression in endocrine cells compared to islet organoids.

However, whether FGF7-induced ACE2 expression specifically impacts islet cells or has a broader influence on other types of organoids or cells has not been determined. Therefore, various organoids and cells known to facilitate SARS-CoV-2 infection, including hESC-derived human lung organoids (HLO), hESC-derived human intestinal organoid (HIO), human umbilical vein endothelial cells (HUVEC), human lung fibroblast (HFL-1) and chondrocytes, were examined. Interestingly, FGF7 treatment significantly increased *ACE2* expression in HIOs and HLOs (Supplementary Fig. 3a, b). However, the difference in *ACE2* expression among HUVECs, HFL-1 cells and chondrocytes treated with FGF7 was marginal and not significant (Supplementary Fig. 3c–e). Furthermore, the *ACE2* gene expression levels in HFL-1 cells, HUVECs and chondrocytes were relatively low (Ct > 32), while those in HLOs and HIOs were notably greater (Ct < 23). To explore the reason for the preferential impact of FGF7 on *ACE2* expression in certain cells but not others, we examined the expression of *FGFRs* (FGFR1, FGFR2, FGFR3 and FGFR4) in each cell type (Supplementary Fig. 3f). Intriguingly, *FGFR2* expression was predominant in both HIOs and HLOs, with FGFR2 mRNA expression relative to GAPDH is around 500 and 350, respectively (Supplementary Fig. 3f). Moreover, *FGFR2* expression in HFL-1 cells, HUVECs and chondrocytes was approximately one order of magnitude lower than that in the organoids. Although FGFR1 was the predominant FGFR gene in HFL-1 cells, HUVECs and chondrocytes, the relatively low ACE2 expression of these cells suggests that there is a less direct connection between FGFR1 and ACE2. In conclusion, we observed a direct correlation between *ACE2* expression and FGFR2 expression, particularly in cells where FGF7 could enhance ACE2 expression, and where FGFR2 expression was predominant.

### FGF7 regulates islet function through modulation of ACE2 expression and activity

ACE2 serves as a crucial receptor for SARS-CoV-2 and also plays a pivotal role as an enzyme that is particularly crucial for insulin secretion and β cells function.^45^ In the FGF7 treated organoids, ACE2 activity was increased by 1.5 times (*p* < 0.05) compared to that in the control group, and this increase was inhibited by FGFR inhibitors (Fig. 3l, m). This finding indicates that the accumulation of ACE2 induced by FGF7 also results in increased ACE2 activity. Subsequently, we assessed insulin secretion in response to glucose stimulation in hESC-derived islet organoids exposed to FGF7 and FGFR inhibitors. The control group exhibited a 1.3-fold greater insulin secretion after treatment with 16.7 mM glucose than after treatment with 2.8 mM glucose (Fig. 3n, black). Interestingly, FGF7 treatment significantly increased insulin secretion by 1.6-fold change in response to high glucose (Fig. 3n, blue). However, in the presence of FGFR inhibitors, ACE2 activity in the islet organoids was reversed, impairing insulin secretion under high glucose conditions (Fig. 3n, purple). These results demonstrated that the FGF7-induced increase in ACE2 expression in β cells promoted insulin secretion under high glucose conditions. Furthermore, islet organoids from G4 showed a significantly reduction in *INS* mRNA expression (*p* < 0.01) and those from G5 showed an increase in *INS* expression (*p* < 0.05) compared with those from G1 (Supplementary Fig. 3g). IF staining of islet organoids revealed that FGF7 significantly decreased the percentage of insulin-positive cells within the islet organoids (Supplementary Fig. 3h, i). In conjunction with the increase ACE2 expression in isolated β cells treated with FGF7, suggests that FGF7 plays a dual role in insulin secretion. Initially, FGF7 enhances insulin secretion by increasing ACE2 in β cells. Simultaneously, the reduction of β cells in FGF7 treated islet organoids may counteract the upregulation of insulin secretion under high glucose stimulation. However, although FGFR inhibitors increased the number of β cells (Supplementary Fig. 3h, i). Blocking ACE2 expression in β cells with FGFR inhibitors (Fig. 3j) impaired insulin secretion (Fig. 3n, purple). The results of gene expression and IF staining of GCG (Supplementary Fig. 3j–l) under FGF7 and/or FGFR inhibitor treatment were consistent with those of INS. The underlying mechanism and downstream pathways of FGF7 and FGFR inhibitors in insulin secretion and the number of endocrine cells present under different conditions require further investigation that is beyond the scope of the current study.

In summary, our findings demonstrated that the activation and inhibition of the FGF7-FGFR signaling pathway can modulate ACE2 expression in islet organoids. FGF7 treatment led to increased ACE2 expression and activity in endocrine hormonal-producing cells, primarily β cells. Conversely, FGFR inhibitors reduce the expression and activity of ACE2, consequently impairing insulin secretion from islet organoids. In conclusion, our study elucidated the role of the FGF7-FGFR pathway in regulating ACE2 expression and insulin secretion in human islet organoids. However, whether the upregulation of ACE2 induced by activation of the FGF7-FGFR pathway would be a beneficial for insulin secretion or potentially promote viral infection during SARS-CoV-2 infection remained uncertain and investigated in the following section.

### FGF7 enhances SARS-CoV-2 viral infection in islet organoids and this effect can be counteracted by FGFR inhibitors

**Fig. 4:**
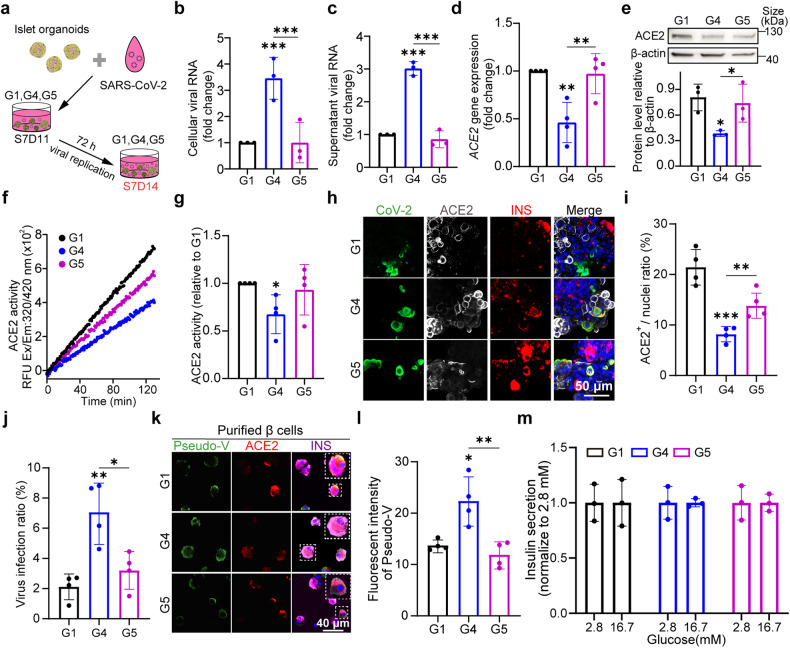
FGF7 exacerbates SARS-CoV-2 infection in islet organoids, thus impairing the insulin secretion. **a** Schematic representation of the methodology used to infect islet organoids from G1, G4 and G5 with SARS-CoV-2: viral replication was tested in the organoids and the virus was released into the supernatant at 72 h post-infection (hpi). Sample collection time at S7D14. **b** qPCR analysis of SARS-CoV-2 replication in the supernatants of G1, G4 and G5 cells. The values are normalized to those of G1 after 3 independent inoculations. **c** qPCR analysis of intracellular viral RNA in islet organoids from G1, G4 and G5. **d** *ACE2* mRNA expression in G1, G4 and G5 at 72 hpi. The values are normalized to G1. **e** Western blot analysis of ACE2 and β-actin in islet organoids at 72 hpi in G1, G4 and G5. **f** The ACE2 enzymatic activity curves of G1, G4 and G5 from 72 hpi islet organoids subjected to a kinetics model for 130 min. **g** ACE2 enzymatic activity (mU) per mg of protein from G1, G4 and G5 islet organoids relative to G1 at 72 hpi. **h** The co-localization of SARS-CoV-2 (CoV-2, green), ACE2 (white), and INS (red) in G1, G4 and G5 from 72 hpi islet organoids. **i** Quantification of (**h**) showing the percentage of ACE2^+^ cells relative to total cells according to the number of nuclei. **j** Quantification of (**h**) showing the percentage of SARS-CoV-2-infected cells ratio in 72 hpi islet organoids from G1, G4 and G5. **k** Tricolor IF co-staining of CoV-2 pseudo-virus spike (pseudo-V, green), ACE2 (red) and INS (purple) in purified β cells from G1, G4 and G5. The image in the top-right corner of the picture represents an enlarged view of the region enclosed within the dashed box. **l** Quantification of (**k**) revealing the fluorescence intensity of pseudo-V in purified β cells from G1, G4 and G5. **m** Insulin secretion of islet organoids at 72 hpi in static GSIS assay. Images are from one representative experiment from 3 to 4 independent experiment. The data are presented as the individual values of 3 to 4 replicates with 3 repeats in each experiment. *p* values were calculated by one-way ANOVA and Tukey’s multiple comparison test, *p < 0.05, **p < 0.01, ***p < 0.001

Prior to infection with live SARS-CoV-2, the islet organoids were pretreated with FGF7 and/or FGFR inhibitors for 3 days to either activate or suppress the FGF7-FGFR pathway. Both intracellular and supernatant samples from the islet organoids were collected for further analysis (Fig. 4a). After 72 hpi, the G4 islet organoids presented higher levels of viral RNA in both the intracellular and supernatant samples than did the G1 organoids (Fig. 4b, c). Conversely, treatment with FGFR inhibitors resulted in a reduction in viral RNA in the islet organoids and supernatant, comparable to the levels observed in G1 islet organoids (Fig. 4b, c). Over time, FGF7 appeared to enhance viral infection within islet organoids and virus release into the supernatant over time, while FGFR inhibitors effectively halted viral replication between 24 hpi and 72 hpi (Supplementary Fig. 4a). Intriguingly, we incidentally discovered that higher glucose levels in the islet organoid culture medium increased viral replication in the supernatant (Supplementary Fig. 4b). These findings suggested that in the context of elevated blood glucose levels, glucose transported from microvascular blood to the islets may exacerbate viral infection in islet Langerhans. Our findings indicate that islet organoids exposed to FGF7 are more vulnerable to SARS-CoV-2 infection, a phenomenon that can be mitigated by FGFR inhibitors.

In contrast to the upregulation of ACE2 induced by FGF7 in the absence of SARS-CoV-2, both the gene and protein levels of ACE2 in G4 islet organoids were significantly reduced after infection (Fig. 4d, e). This reduction might be attributed to increased SARS-CoV-2 viral accumulation and replication, potentially resulting in the consumption of more ACE2 receptors. A previous study reported a similar downregulation of ACE2 after SARS-CoV-2 infection.^46^ Consequently, the enzymatic activity of ACE2 in FGF7-treated islet organoids decreased at 72 hpi (Fig. 4f, g). Notably, compared to uninfected islet organoids, SARS-CoV-2 infection significantly reduced the enzymatic activity of ACE2 in the islet organoids progressively over time (Supplementary Fig. 4c, d). IF staining indicated that, compared to the G1, FGF7 combined with SARS-CoV-2 significantly reduced the number of ACE2^+^ cells in the islets (Fig. 4h, G4 group). Moreover, FGFR inhibitors reversed the FGF7-induced reduction in ACE2 expression during viral infection (Fig. 4h, G5 group). In the presence of FGF7, the ACE2 ratio in islets decreased from 20 to 9% following exposure to SARS-CoV-2 but rebounded to 15% after FGFR inhibitor treatment (Fig. 4i). IF staining also demonstrated that the viral N-protein infection ratio in FGF7-treated islets was ~3 times greater than that in G1 and G5 islets (Fig. 4j). Although some cells exhibited double positivity for the pancreatic hormones INS and the viral N protein, most SARS-CoV-2-infected cells lacked hormone expression (Supplementary Fig. 4e). Previous research has demonstrated that SARS-CoV-2 can induce the dedifferentiate of insulin-positive β cells into NKX6.1^+^ progenitor.^47^ In our study, co-staining of the SARS-CoV-2 N protein, NKX6.1, and insulin indicated that SARS-CoV-2 infection resulted in a decrease in insulin signaling, while NKX6.1 signaling was maintained (Supplementary Fig. 4f). In summary, FGF7 enhanced viral infection in islet organoids while simultaneously decreasing ACE2 levels in response to SARS-CoV-2 infection.

The observation of reduced ACE2 levels during live SARS-CoV-2 infection led to rare co-expression of ACE2 and live virus making it challenging to verify the direct binding of SARS-CoV-2 to islet β cells through ACE2. The spike protein is present on the surface of SARS-CoV-2 and facilitates viral infection. SARS-CoV-2 pseudo-virus has been extensively used to assess the binding affinity of the spike protein for various types of cells expressing SARS-CoV-2-related receptors, especially ACE2.^48,49^ To assess whether SARS-CoV-2 binds to islets via ACE2 and whether FGF7 influences the binding rate, SARS-CoV-2 pseudo-virus was incubated with purified β cells. It is not surprising that the majority of cells permissive to pseudo-virus (green) infection were also co-stained with ACE2 (red, Fig. 4k). FGF7-induced upregulation of ACE2 (red) expression significantly intensified GFP (green) signaling in β cells (purple) compared to that in the control cells (Fig. 4k, l). However, FGFR inhibitors prevented the FGF7-induced binding of pseudo-viral particles to β cells (Fig. 4k, l). These results suggest that the infection of β cells by the virus predominantly relies on ACE2 expression, suggesting that COVID-19 patients with higher FGF7 concentrations may experience more severe damage to their islet β cells. The efficiency of pseudo-virus binding aligns with the intracellular viral RNA level and the presence of the N protein presence in islet organoids following the addition of FGF7.

The efficiency of pseudo-virus binding was also assessed in purified α and δ cells. Unexpectedly, IF staining revealed a notably greater binding rate (50%) of the SARS-CoV-2 spike protein to the α cells in the G4 group. FGFR inhibitors effectively reduced the spike-binding rate in the G5 subset of α cells (Supplementary Fig. 4g). Therefore, the significantly greater of SARS-CoV-2 spike-binding rate in α cells suggested the involvement of other SARS-CoV-2-related receptors and proteases in addition to ACE2. In the G1 group, δ cells rarely exhibited ACE2 signals, and pseudo-viruses seldom bound to SST-positive cells in the G1 group. However, we found pseudo-virus binding to δ cells in our FGF7-treated (G4) and alofanib-inhibited (G5) groups (Supplementary Fig. 4h). Intriguingly, the δ cells that interacted with the pseudo-virus did not express ACE2 (Supplementary Fig. 4h, white arrowhead). Approximately 18% of the δ cells in the FGF7-treated group (G4) interacted with the pseudo-virus, suggesting that FGF7 upregulated other SARS-CoV-2 receptors (not ACE2) expressed on the δ cells. While the lower binding rate of pseudo-virus to δ cells was likely due to the absence of ACE2. Our findings suggest that FGF7 may enhance viral infection and replication primarily in β cells rather than in α and δ cells through ACE2.

SARS-CoV-2 infection significantly decreased the total insulin content 7 times in the hESC-derived islets (Supplementary Fig. 4i). Consequently, the insulin secretion response to a high glucose pulse was nearly diminished at 72 hpi, regardless of FGF7 or FGFR inhibitor treatment (Fig. 4m). In conjunction with the sharp decrease in ACE2 activity in islet organoids at 72 hpi (Supplementary Fig. 4c, d), total insulin and ACE2 activity were compromised by SARS-CoV-2. To assess whether FGF7 and FGFR inhibitors can assist insulin secretion before the virus damages insulin-secreting β cells, we evaluated glucose stimulation insulin secretion (GSIS) in islet organoids at 24 hpi. GSIS was maintained in G1 and G4 islets (Supplementary Fig. 4j); Accordingly, the ACE2 enzymatic activity in G1 and G4 islets was similar at 24 hpi (Supplementary Fig. 4c, d). However, G5 islets with lower ACE2 activity did not respond to the high-glucose pulse at both 24 and 72 hpi (Figs. 4m and S4j). Our results suggest that FGF7 activation of the FGF7-FGFR signaling pathway increases ACE2 expression, consequently enhancing viral infection and replication in islet organoids. Because β cells are more susceptible to ACE2 overexpression induced by FGF7, they become the primary target cells infected and destroyed by the virus. Accordingly, GSIS considerably decreased when islet organoids were exposed to SARS-CoV-2, and FGF7 further reduced the insulin concentration. Although FGFR inhibitors counteracted the excessive viral infection induced by FGF7 and reduced ACE2 consumption, FGFR inhibitors did not improve GSIS. In summary, our findings suggest that FGF7 exacerbates SARS-CoV-2 infection in islet organoids through ACE2. While GSIS was sustained for 24 hpi in FGF7-treated islet organoids, the damage to β cells eventually led to a diminished GSIS. Ultimately, FGFR inhibitors reversed FGF7-induced SARS-CoV-2 infection but did not enhance insulin secretion.

### The serum and pancreatic FGF7 levels varied under SARS-CoV-2 infection in vivo

**Fig. 5:**
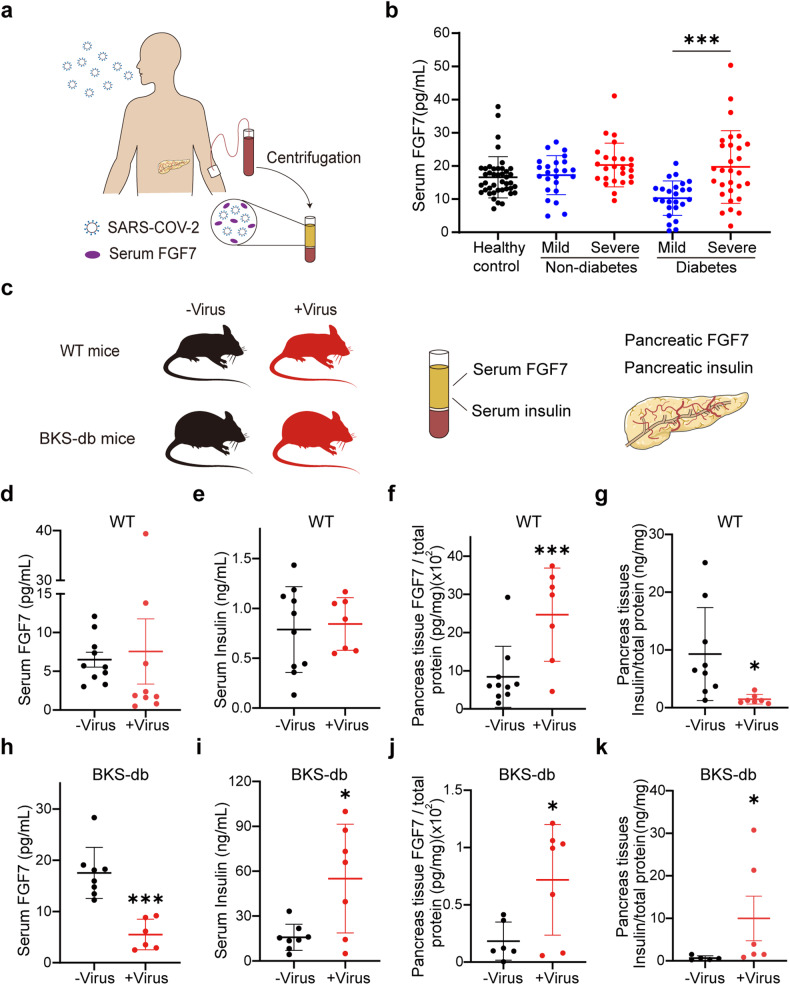
Measurement of FGF7 in the serum and pancreas during SARS-CoV-2 infection in vivo. **a** Schematic representation of clinical serum samples collected from COVID-19 patients with diabetes or without diabetes. **b** Serum FGF7 concentrations in nondiabetic or diabetic patients with mild or severe clinical symptoms. *n* = 30 patients each group. *n* = 44 healthy individuals were recruited as controls. **c** Schematic of the experiment performed with wild-type (wt) mice and diabetic (BKS-db) mice model. **d**–**g** Fluctuation in the serum FGF7 concentration, serum insulin concentration, pancreatic FGF7 concentration and pancreatic insulin concentration in wt mice before and 7 days after SARS-CoV-2 inoculation. **h**–**k** Fluctuation in the serum FGF7 concentration, serum insulin concentration, pancreatic FGF7 concentration and pancreatic insulin concentration in BKS-db mice before and 7 days after SARS-CoV-2 inoculation. The data are represented as the values of individual clinical samples or mouse samples. *p < 0.05, ***p < 0.001 according to an unpaired two-tailed *t*-test

To investigate the impact of SARS-CoV-2 infection on FGF7 levels and insulin secretion in vivo, a retrospective clinical study and animal experiments were conducted. A total of 120 COVID-19 patients were recruited from Wuhan Jinyintan Hospital between January 2023 and June 2023 and blood serum samples were collected for ELISA (Fig. 5a). The severity of COVID-19 was classified based on the clinical criteria of the WHO,^50^ and the patients were divided into four groups: 30 nondiabetic patients with mild COVID-19, 30 nondiabetic patients with severe COVID-19, 30 diabetic patients with mild COVID-19 and 30 diabetic patients with severe COVID-19. Additionally, 44 healthy volunteers served as uninfected controls. Among the COVID-19 patients without a history of diabetes, there were no significant differences in the serum FGF7 levels among the uninfected (20.30 ± 8.85 pg/ml), mild (17.20 ± 5.88 pg/ml) and severe (20.27 ± 6.59 pg/ml) patients (Fig. 5b). However, in diabetic patients with COVID-19, the serum FGF7 concentration increased with severity (mild vs. severe: 10.28 ± 5.20 pg/ml vs. 19.70 ± 10.95 pg/ml, *p* = 0.0003). The serum samples from COVID-19 patients exhibit the heterogeneous FGF7 concentrations in each individual, but FGF7 levels increase with the severity of COVID-19 in diabetes, implying a potential relationship between FGF7 and diabetes.

A ridge plot was generated using an online tool (http://www.sangerbox.com/home.html) to visualize the distribution pattern of FGF7 in the different groups. The distribution of FGF7 in diabetic patients with severe COVID-19 symptoms appeared wider compared to other groups (Supplementary Fig. 5a). However, the distribution of FGF7 in diabetic patients with mild symptoms was narrow and shifted to the left (indicating a lower FGF7 concentration). Receiver operating characteristic (ROC) curve analysis of the serum FGF7 concentration indicated an area under the curve (AUC) of 0.68 ± 0.05 for COVID-19 patients without diabetes, suggesting lower diagnostic accuracy (Supplementary Fig. 5b). However, for COVID-19 patients with diabetes, the AUC was 0.79 ± 0.06, indicating a more accurate diagnostic result (Supplementary Fig. 5c). The determined cutoff value based on the sensitivity and specificity for distinguishing mild and severe cases was 13.7 ng/ml, which could be considered as a serum FGF7 indicator for distinguishing severe from mild COVID-19 comorbid with diabetes. The correlation between serum FGF7 levels and age was also examined. A horizontal dashed line was drawn at an FGF7 concentration of 13.7 ng/ml. A plot of the serum FGF7 concentration against age showed that 72% of nondiabetic patients with mild COVID-19 and 79% of nondiabetic patients with severe symptoms were above the 13.7 ng/ml with more elderly patients in the severe group. Among those with diabetes, 20% had mild symptoms, and 73% had severe symptoms. FGF7 values were distributed above the dotted line (Supplementary Fig. 5d, red dots). These findings suggested that a serum FGF7 concentration of 13.7 ng/ml is a reliable threshold for indicating the severity of the COVID-19 patients’ comorbid diabetes. The COVID-19 patients with preexisting diabetes and severe respiratory symptoms included more elderly patients and had higher serum FGF7 concentration. However, the serum FGF7 concentration may not reflect the local distribution of FGF7 in tissues during SARS-CoV-2 infection. Therefore, diabetic (db) and wild-type (wt) mice were inoculated with SARS-CoV-2 to study the in situ variations in FGF7 levels during viral infection.

Eight-weeks-old db mice and wt mice were inoculated with the β strain of SARS-CoV-2, and the major viral target organs and blood samples were collected after 1 week (Fig. 5c). Mice infected with SARS-CoV-2 exhibited mild symptoms, characterized by slight weight loss for 3 days followed by weight gain indicative of mild symptoms (Supplementary Table 7). As in nondiabetic patients with mild COVID-19 symptoms, there was no significant alteration in serum FGF7 and insulin levels compared to the control (Fig. 5d, e). Whilst, homogenates of the pancreatic tissue from infected wt mice presented a significantly greater concentration of in situ FGF7 than did the uninfected control tissue (Fig. 5f) while the insulin concentration in pancreatic tissue decreased (Fig. 5g). This aligns with our observations in hESC-derived islet organoids, where FGF7 led to decreased insulin contents in the islets. In db mice, serum FGF7 levels significantly decreased under exposure to SARS-CoV-2 (Fig. 5h), mirroring the decrease seen in diabetic patients with mild COVID-19 symptoms. Concurrently, serum insulin levels in db mice increased with SARS-CoV-2 infection (Fig. 5i). Clinical reports have indicated that individuals with diabetes mellitus under COVID-19 often exhibit insulin resistance, accompanied by increased insulin concentrations in the blood.^46^ In the homogenate of pancreatic tissue from db mice, in situ FGF7 levels increased after SARS-CoV-2 infection (Fig. 5j). The lower pancreatic insulin concentration (Fig. 5k) in db mice than in wt mice might be attributed to the surrounding fatty tissue near the intestine, making pancreatic collection challenging.

The variation in FGF7 concentrations in SARS-CoV-2-targeted organs (the liver, lung and intestine) of both wt and db mice were assessed as well. In tissues collected from wt mice, the liver exhibited a greater FGF7 concentration (Supplementary Fig. 5e, ~10,000 pg/mg protein) than did the lung (Supplementary Fig. 5f, ~4000 pg/mg protein) and the intestine (Supplementary Fig. 5g, less than 10 pg/mg protein), suggesting a heterogeneous in vivo distribution of FGF7. However, the FGF7 concentrations in these organs were unaffected by SARS-CoV-2. In tissues collected from db mice, the concentrations of FGF7 in the liver (Supplementary Fig. 5h) and intestine (Supplementary Fig. 5j) remained unchanged, but the FGF7 level decreased in the lung (Supplementary Fig. 5i) during viral infection. Our clinical and animal data revealed a heterogeneous in vivo distribution of FGF7, inconsistency between serum and tissue FGF7 levels, and an increase in the FGF7 concentration in the pancreas during SARS-CoV-2 infection. These findings imply a potential correlation between the upregulation of FGF7 and pancreatic function.

## Discussion

Considered collectively, our results indicate that FGF7 primarily interacts with FGFR2 to upregulate ACE2 expression and activity in islet organoids, especially in β cells, thereby aggravating SARS-CoV-2 infection. Treatment with FGFR inhibitors efficiently block the FGF7-induced ACE2 upregulation which prevents SARS-CoV-2 infection.

The finding of FGF7 in regulating ACE2 expression in islets is not surprising. Within the human pancreas, FGF7 is secreted by pancreatic stromal tissue and plays a pivotal role in pancreatic regeneration and islet implantation.^51,52^ Our animal experiments detected an increase in pancreatic FGF7 levels in both db and wt mice under SARS-CoV-2 infection compared to uninfected animals (Fig. 5f, j). However, FGF7 levels in other tissues (e.g., liver, lung and intestine) remained unchanged or slightly decreased during SARS-CoV-2 infection. Utilizing hESC-derived intestinal and lung organoids, we also found FGF7 upregulates ACE2 expression in intestinal and lung organoids. However, due to the relatively low FGF7 levels in intestinal tissue and its decrease in lung tissue infected by SARS-CoV-2, concerns about FGF7’s influence in these organs may be less critical. Our results suggest that SARS-CoV-2 specifically upregulates pancreatic FGF7 concentration relative to other tissues, implying a close correlation between FGF7 and the pancreas function under COVID-19. Besides widely restored in the tissue, plasma FGFs have emerged as potential diagnostic biomarkers and therapeutic targets for SARS-CoV-2 infection.^13,14^ The most widely investigated plasma FGFs under SARS-CoV-2 infections are FGF2 (FGF basic),^13,14^ FGF19,^15^ FGF21^15^ and FGF23.^15,16^ However, the FGF7 levels in the circulation system under COVID-19 has not been investigated to date. Our clinical retrospective study found the higher serum FGF7 could serve as a promising biomarker for assessing COVID-19 severity in diabetes. Through our analysis with ROC curves, we determined the cutoff value of FGF7 to be 13.7 pg/ml between severe and mild COVID-19 patients, to predict the infection severity.

An increasing number of publications suggest that inhibiting ACE2 expression in host cells, especially the nasal epithelial cells,^53,54^ is a promising strategy to prevent SARS-CoV-2 infection. However, suppressing ACE2 in the pancreas may lead to an imbalance in insulin secretion. Thus, while reducing ACE2 expression seems promising for blocking the virus in host cells, its regulation in human islets requires careful consideration. Recent evidence indicates a significant decrease in ACE2 levels in the metabolic system organs (e.g., kidney and heart) of COVID-19 patients, leading to metabolic sequelae.^46,55^ Therefore, restoring and upregulating ACE2 expression and activity could benefit patients facing metabolic challenges associated with COVID-19. Metformin, an anti-diabetes medication known to increase ACE2, has shown potential benefits for patients with COVID-19.^56^ Our study revealed the significance of ACE2 in the β cells in the response to glucose stimulation. FGF7 enhanced ACE2 expression while FGFR inhibitors counteracted the FGF7-induced ACE2 upregulation in islets especially in β cells. Although the number of β cells increased in FGFRi-treated organoids, the absence of ACE2 resulted in nonresponsive GSIS in FGFR inhibitor-treated organoids. This finding is in consistent with the previous study that FGF21 increased insulin mRNA and protein levels without potentiating glucose-induced insulin secretion from the islets of healthy rats.^57,58^ Overall, FGF7-enhanced ACE2 functions primarily as an enzyme that promotes insulin secretion from islet organoids without exposure to the SARS-CoV-2. However, during SARS-CoV-2 infection, the enhanced ACE2 expression induced by external stimulation provides additional viral binding sites. Over time, ACE2 is occupied and consumed by the virus, after which its enzymatic activity is disrupted.

The limitation of current study is the precise downstream pathway through which FGF7 regulate ACE2 remains unclear. FGF7 interacts with primarily FGFR2 and FGFR1 to activate the downstream pathways. Our study found silencing of FGFR1 and FGFR2 in pancreatic cells indicated FGFR2 directly determines ACE2 expression. The combination of FGFR1 and FGFR2 inhibitors (PD and alofanib) works better than the solely applied FGFR2 inhibitor (alofanib only). But the underlying mechanism and pathways between FGF7/FGFR and ACE2 need to be further investigated.

In summary, this study highlights the significance of FGF7, a ubiquitous present paracrine small molecule, in enhancing ACE2 expression and activity during pancreatic development and in functional endocrine cells (α and β cells). During SARS-CoV-2 infection, the increase in ACE2 expression induced by FGF7 provides additional viral binding sites, facilitating faster viral invasion and replication in the cells. FGFR inhibitors can mitigate FGF7-induced viral infection. Thus, the FGF7-FGFR2 regulatory pathway is emerging as a robust mechanism influencing ACE2 expression in the islets, particularly in β cells. On the basis of our findings and conclusions, we hypothesize that inhibiting the FGF7-FGFR2 pathway to reduce ACE2 expression could help prevent viral infection thereby ameliorate COVID-19.

## Materials and methods

For details on the information presented this section, please also see the Supplementary figures and tables.

### hESC maintenance and differentiation of hESC into pancreatic islets

The human embryonic stem cell (hESC) line H1 (WiCell Research Institute) was maintained in the mTeSR1 medium (StemCell Technologies, Cat#85850) in a 6 well plate at 37 °C, 5% CO_2_, and 100% humidity. The medium was changed every day and the cells were passaged every 5–6 days. The hESCs were differentiated into pancreatic islet organoids based on previously published protocols^34,42,59^ with slight modifications (details are provided in the Supplemental experimental procedures). 2D cultured cells were dissociated and aggregated into 3D cell aggregates in the ultra-low attachment 6-well plates (Corning, Cat# 3471) at S5D3 following previously published methods.^60,61^ The 6-well plates were placed on a shaker for the S6 and S7 differentiation. The endocrine cells self-aggregated into clusters within 24 h, whereas progenitors, exocrine cells and other unknown cells remained in the supernatant and were removed from the culture medium. The culture medium was refreshed every other day during S6 and S7. The differentiation protocols and efficiency are detailed in Supplementary Table 1.

### Experimental grouping for FGF7 and FGFR inhibitors treatments

To assess the impact of FGF7 on ACE2 expression during both the early and late developmental stages, we introduced FGF7 (50 ng/ml), and FGFR1 inhibitor (PD166866, MCE, Cat#HY-101296, 5 μM), and an FGFR2 inhibitor (alofanib, Selleckchem, Cat#RPT835, 5 μM) during S5 (endocrine progenitors), or from S7D7 to S7D14, respectively. Our study comprised five main experimental groups: Group 1 (G1) served as the control without additional modifications to the differentiation protocol. Group 2 (G2) received 50 ng/ml FGF7 at S5. Group 3 (G3) was supplied with both 50 ng/ml FGF7 and FGFRi (5 μM alofanib and 5 μM PD166866) at S5. Group 4 (G4) received 50 ng/ml FGF7 from S7D7 to S7D14. Lastly, Group 5 (G5) was treated with both 50 ng/ml FGF7 and 5 μM FGFRi (5 μM alofanib and 5 μM PD166866) from S7D7 to S7D14. FGFs and FGFRs used in this study were summarized in Supplementary Table 2.

### Small interfering (si) RNA (siRNA) and transfection

Small interfering RNA (siRNA) targeting FGFR1 and FGFR2 was designed and chemically synthesized by Tsingke Biotech (Guangzhou, China). The sequences are listed in Supplementary Table 3. Pancreatic endocrine progenitors were dissociated and seeded at a concentration of 5 × 10^5^ cells/ml in 24-well plates, and a 2 μl of siRNA mixture (20 pmol/μl) was transfected into the cells using Lipofectamine RNAiMAX (Thermo Fisher Scientific, 13778030) for 6 h. After transfection, the transfection solution was removed, and stage-specific culture medium was added to the culture for 24 h. The cells were lysed for RNA extraction and subsequent qPCR analysis.

### Magnetic enrichment of α and β cells

Islet organoids from groups G1, G4 and G5 were collected on S7D14 and magnetically enriched following published protocols with slight modifications^41,42^ (also see Supplemental experimental procedures in Supplementary Data). Purified α, purified β and α-β- cells (remaining cells) were used to prepare mRNA and total proteins and IF imaging of cells-seeded on coverslips; the samples were subjected to distribution and concentration analyses of major SARS-CoV-2 receptors and proteases.

### SARS-CoV-2 pseudo-virus preparation and infection

For the infection experiment, purified α, purified β and α-β- cells (5 × 10^5^ cells/ml) were combined with SARS-CoV-2 pseudo-virus (diluted 1:10 with cell suspension) and seeded onto Matrigel-coated glass coverslips (12 mm diameter; Cellvis, Cat#51210) for 2 h. The cells infected with pseudo-virus were allowed to adhere to the coverslip. Subsequently, the cells were washed with DPBS, and the culture medium (S7 medium) was replaced. After 72 h, the coverslips were collected for further analysis. Cells that underwent the same procedure but without pseudo-virus infection were used as a control.

### SARS-CoV-2 infection of islet organoids

SARS-CoV-2 was isolated from COVID-19 patients in Guangdong, China, by the Guangdong Provincial Center for Disease Control and Prevention, China. All work involving live SARS-CoV-2 was performed at the Biosafety Level 3 Laboratories of Guangzhou Customs District Technology Center (P3 lab, Bioland, Guangzhou, China).

SARS-CoV-2 infection of hESC-derived organoids was performed in S7 culture medium at an multiplicity of infection (MOI) = 1 for 2 h at 37 °C. Then, the islet organoids were washed three times with DPBS. Fresh S7 islet organoid culture medium was added, and the mixture was incubated at 37 °C for 24 to 72 h. At the end of the experiments, the organoids were collected for further investigation. The samples were disinfected in Triton X-100 (1% final concentration) for 1 h before release from the P3 laboratory.

### Viral RNA isolation and RT‒qPCR

Viral RNA was isolated from both the cells and supernatants of infected islet organoids were isolated following previously published methods, with slight modifications.^47^ Islet organoids infected with SARS-CoV-2 were lysed in 600 μl of RLT buffer supplemented with 1% β-mercaptoethanol, mixed with a pipette until complete lysis occurred, and stored at −20 °C or −80 °C until further isolation using the QIAGEN RNeasy Plus Mini Kit (catalog no. 74106). For viral RNA in the supernatant, 140 μl of supernatant was mixed thoroughly with 560 μl of AVL buffer and stored at −20 °C until further isolation using a QIAGEN Viral RNA Mini Kit (catalog no. 52906) following the manufacturer’s instructions. Viral gene amplification was performed with primer sets specific for the RdRp gene using TaqMan Fast Virus 1-Step Master Mix (Thermo Fisher Scientific, catalog no. 4444436) and a CFX96 Touch Real-Time PCR Detection System (Bio-Rad). A standard curve was generated to calculate the number of copies of viral RNA in the supernatant. GAPDH was used as an endogenous control to calculate relative viral gene expression in the cells. All the reactions were performed in triplicate.

Primer sets^62^:

RdRp gene

Forward primer: 5’-GTGAAATGGTCATGTGTGGCGG-3’

Reverse primer: 5’-CAAATGTTAAAAACACTATTAGCATA-3’

Probe: 5’-FAM-CAGGTGGAACCTCATCAGGAGATGC-BHQ-3’

### ACE2 enzymatic activity assay

The enzymatic activity of ACE2 in islet organoids was measured using an ACE2 activity assay kit (Fluorometric) (Abcam, ab273297), which leverages the ability of active ACE2 to cleave a synthetic MCA-based peptide substrate, resulting in the release of a free fluorophore. Initially, islet organoids were collected and lysed in ACE2 lysis buffer kept on ice for 30 min. The protein concentration in the lysate was determined using a BCA Protein Assay Kit (Beyotime, P0010). Subsequently, the lysates were mixed with the ACE2 substrate in a 96-well black plate with a flat bottom. Fluorescence kinetics were measured using a multi-plate reader (Biotek synergy neo2) at an excitation wavelength of 320 nm and an emission wavelength of 420 nm for 2 h. The standard curve and calculations were performed according to the ACE2 activity assay kit protocol. Similarly, the ACE2 activity in islet organoids infected with SARS-CoV-2 was evaluated.

### Glucose-stimulated insulin secretion (GSIS)

The hESC-derived islet organoids collected at S7 were subjected to GSIS. The islet organoids were dissociated with TrypLE solution for ~10 min to obtain small cell clusters. The cell clusters were re-plated on the Matrigel-coated 48-well cell culture plates at a density of 5 × 10^5^ cells/well. The cells were maintained in S7 culture medium for 24 h or until they reached 80% confluence before glucose stimulation. The cells were washed twice with Krebs ringer bicarbonate (KRB buffer, pH 7.2, composed of 115 mM NaCl, 5 mM KCl, 1 mM MgCl_2_, 2.5 mM CaCl_2_, 24 mM NaHCO_3_, 10 mM HEPES, and 10% BSA) and pre-incubated with 2.8 mM glucose in KRB for 60 min at 37 °C to balance the islets at a low glucose concentration. Next, the islets were incubated sequentially with KRB supplemented with low glucose (2.8 mM) or high glucose (16.7 mM) for 30 min. After incubation, the supernatant was collected and centrifuged to remove the cell debris. To measure the intracellular insulin content of the islet organoids, the cells were lysed using RIPA buffer (pH 7.4) on ice, followed by brief sonication. The resulting supernatant and lysed cells were stored at −20 °C, and the insulin concentration was quantified within 1 week using a human insulin enzyme-linked immunosorbent assay (ELISA, ALPCO; 80-Insulin). GSIS assays of SARS-CoV-2-infected islet organoids were conducted as described above. The supernatant and cell lysate were disinfected with Triton X-100 (1% final concentration) for 1 h at room temperature to inactivate SARS-CoV-2.

### Clinical sample collection

This experiment was approved by the Institutional Review Board (IRB) of Wuhan Jinyintan Hospital. Clinical samples were collected from COVID-19 patients admitted to Wuhan Jinyintan Hospital (Wuhan, China) between January 2023 and June 2023. The severity of COVID-19 disease was classified based on clinical criteria from the WHO.^50^ For the quantitative determination of plasma FGF7 levels in COVID-19 patients, a series of fasting blood samples from 30 nondiabetic patients with mild COVID-19, 30 nondiabetic patients with severe COVID-19, 30 diabetics patients’ mild, and 30 diabetic patients with severe COVID-19 who have laboratory-confirmed SARS-CoV-2 infection were collected. For comparison, blood samples from 44 healthy volunteers were collected as uninfected controls. To obtain serum from whole blood, the samples were centrifuged at 1000 × *g* at 4 °C for 10 min. The serum was separated from blood cells and collected for further investigation. All the serum samples were disinfected at a temperature of 56 °C for 30 min. Serum FGF7 was assessed with a Human KGF/FGF7 Quantikine ELISA Kit (R&D Systems, DKG00) following the manufacturer’s protocol.

### Animal experiments

All animal experimental procedures were conducted at the Biosafety Level 3 Laboratories of Guangzhou Customs District Technology Center and were approved by the institutional animal care and use committee. Eight-week-old BKS-db (BKS-Leprem2Cd479/Gpt, strain no. T002407 Genotype (Lepr) KO/KO, GemPharmatech, Guangzhou, China) male mice (30–40 g, fasting blood glucose above 11.1 mmol/l) were used as a model of type 2 diabetes. In addition, the wild-type (wt) mice (genotype (Lepr) wt/wt, 20–25 g) were used as the normal control group. BKS-db and wt mice were assigned randomly to the SARS-CoV-2 infection or control group. Mice were inoculated via the intranasal route with 5 × 10^4^ plaque-forming units (PFUs) in 100 μl of DPBS. After 7 days, the mice were euthanized and the pancreas, lung, liver, intestine and serum were collected for quantification of the FGF7 concentration using ELISA kit. The weight was measured daily to monitor the recovery of the infected mice. The tissues were homogenized and disinfected with Triton X-100 (1% final concentration) for 1 h at room temperature to inactivate SARS-CoV-2. The FGF7 concentration in the homogenates was analyzed via a Mouse Keratinocyte Growth Factor (KGF/FGF-7) Elisa Kit (SAB Signalway Antibody, EK11257).

### qPCR analysis

In our study, we utilized standard techniques for mRNA isolation and qPCR. The detailed experimental procedures can be found in the Supplementary Data. The primer sequences specific for SARS-CoV-2 receptors, proteases, and genes associated with islet development and maturation are listed in Supplementary Table 3.

### Western blotting

Total protein was isolated from cells subjected to different treatments with RIPA buffer (Beyotime, Cat# P0013B) containing a protease inhibitor mixture (Beyotime, Cat# P1006). The protein concentration in the lysates was quantified using BCA protein assay kit (Beyotime, Cat# P0010). Equal amounts (40 μg) of total protein were loaded onto 4–20% precast polyacrylamide gels (GenScript, Cat#M42015C). The antibodies and dilutions used in the study are summarized in Supplementary Table 4. A protein ladder (GenScript, Cat# M00624-250) was used to determine the band size.

### Immunofluorescence staining

The 2D cultured or replated cells were fixed in 4% (w/v) paraformaldehyde (PFA) for 30 min at room temperature, incubated with DPBS and stored at 4 °C for further staining. 3D-suspended islet organoids, subjected to various treatments (±virus, ±pseudovirus, ±FGF7 or ±FGFRi) were rinsed with DPBS twice and subsequently fixed in 4% PFA for 30 min at room temperature. After fixation, the organoids were rinsed three times with DPBS and incubated overnight in 30% sucrose solution at 4 °C. Then, the organoids were submerged in OCT solution (Biosharp, Cat# BL557A), flash frozen in liquid nitrogen and stored at −80 °C for cryo-sectioning. The frozen samples were sliced into 7 μm sections using a freezing microtome (Minux®FS800, RWD, China) and collected on Superfrost Plus slides (CITOTEST, Cat# 80312-3161). The sections were fixed shortly in 4% PFA and stored at −20 °C before staining. Live SARS-CoV-2 infected islet organoids were fixed with 4% PFA overnight at 4 °C and subjected to whole mounting and IF staining. The detailed IF staining protocol and antibodies used are described in the Supplemental procedure section and Supplementary Table 4. The cellular fluorescence intensity was measured using ImageJ, and the corrected total cell fluorescence (CTCF) was calculated with the following formula: CTCF = integrated density − (area of selected cell × mean fluorescence of background reading). The specific percentage of positive cells was calculated as the number of positive cells divided by the number of nuclei.

### Bulk RNA sequencing

Endocrine progenitors (G1, G2 and G3) were lysed using TRIzol (Thermo Fisher, Cat# 15596018) and flash frozen in liquid nitrogen for bulk RNA-seq analysis (three typical samples were taken from each group for mapping analysis). Quality testing, database construction and RNA sequencing were subsequently performed by Beijing Genomics Institution (BGI, Shenzhen, China). A gene expression matrix (tpm) of 60286 genes at S5D3 (G1, G2 and G3, 3 samples for each group) was generated. A complete ACE2-related network with 68 genes was constructed by Wicik et al.^44^ and referenced in our data analysis. The initial exploratory analysis included heatmaps, volcano plots and bubble plots, all of which were annotated with variables used in expression modeling (Complex Heatmap v2.10.0, ggplot2 v3.3.6, ggrepel v0.9.1). The heatmaps were generated by comparing the 68 genes in the published ACE2 interaction network with our sequencing results. The genes with significant differences were annotated. A heatmaps of the genes with significantly differential expression (up- or downregulated; *p* < 0.05 and log2(FC) > |1|) was also generated. The genes marked in the heatmaps are indicated in the volcano plots according to their significant up- (red) or downregulation (blue). The bubble plots reveled the top 7 pathways significantly enriched in the KEGG pathways. All bulk RNA sequencing figures were generated using R, version 4.1.3.

### Statistical analysis

All the experiments were repeated at least three times. The data are presented as the means ± standard deviation (SD). Statistical analysis was performed using an unpaired Student’s *t* test for comparisons between two groups and one-way ANOVA and Tukey’s multiple comparison test for comparisons among more than two groups. Immunofluorescence imaging was performed on Z-stacks acquired with a confocal microscope from at least five (*n* = 5) independent biological samples. For clinical samples, we generated a receiver operating characteristic (ROC) curve, with the optimal cutoff point being the FGF7 concentration where the maximum (Sensitivity% + Specificity% − 1) was achieved. This study aimed to determine the clinical value of FGF7 in assessing the severity of pneumonia. All of the statistical analyses in this study were performed with GraphPad Prism 8 software. A *p* value less than 0.05 is considered to indicate statistical significance.

### Supplementary information

Supplementary Materials

## Data Availability

The corresponding authors will provide the study’s data upon reasonable request. The bulk RNA-seq data can be viewed in NODE (https://www.biosino.org/node) by pasting the accession (OEP005097) into the text search box or through URL: https://www.biosino.org/node/project/detail/OEP005097.

## References

[1] Aleem, A., Akbar Samad, A. B. & Vaqar, S. Emerging variants of SARS-CoV-2 and novel therapeutics against coronavirus (COVID-19). *In: StatPearls [Internet]* (2023). 34033342

[2] Oudit GY, Wang K, Viveiros A, Kellner MJ, Penninger JM (2023). Angiotensin-converting enzyme 2 at the heart of the COVID-19 pandemic. Cell.

[3] Lu Y (2022). SARS-CoV-2 down-regulates ACE2 through lysosomal degradation. Mol. Biol. Cell.

[4] Li M-Y, Li L, Zhang Y, Wang X-S (2020). Expression of the SARS-CoV-2 cell receptor gene ACE2 in a wide variety of human tissues. Infect. Dis. Poverty.

[5] Beyerstedt S, Casaro EB, Rangel ÉB (2021). COVID-19: angiotensin-converting enzyme 2 (ACE2) expression and tissue susceptibility to SARS-CoV-2 infection. Eur. J. Clin. Microbiol. Infect. Dis..

[6] Takabayashi T, Yoshida K, Imoto Y, Schleimer RP, Fujieda S (2022). Regulation of the expression of SARS-CoV-2 receptor angiotensin-converting enzyme 2 in nasal mucosa. Am. J. Rhinol. Allergy.

[7] Martens K, Vanhulle E, Viskens AS, Hellings PW, Vermeire K (2023). Fluticasone propionate suppresses the SARS-CoV-2 induced increase in respiratory epithelial permeability in vitro. Rhinology.

[8] Smyth JS (2023). Farnesoid X receptor enhances epithelial ACE2 expression and inhibits virally induced IL-6 secretion: implications for intestinal symptoms of SARS-CoV-2. Am. J. Physiol. Gastrointest. Liver Physiol..

[9] Hui Q, Jin Z, Li X, Liu C, Wang X (2018). FGF family: from drug development to clinical application. Int. J. Mol. Sci..

[10] Xie Y (2020). FGF/FGFR signaling in health and disease. Signal Transduct. Target Ther..

[11] Cuevas P (2021). Dobesilate an old drug as a possible new treatment option for COVID-19 infection. Int. J. Med. Rev. Case Rep..

[12] Cuevas, P., Manquillo, A., Guillen, P. & GIménez-Gallego, G. Fibroblast growth factor: a target for Covid-19 infection. *Int. J. Med. Rev. Case Rep.* **4**, 122–125 (2020).

[13] Gupta A (2022). SARS-CoV-2 infection-induced growth factors play differential roles in COVID-19 pathogenesis. Life Sci..

[14] Xu ZS (2020). Temporal profiling of plasma cytokines, chemokines and growth factors from mild, severe and fatal COVID-19 patients. Signal Transduct. Target Ther..

[15] Soares-Schanoski A (2022). Asymptomatic SARS-CoV-2 infection is associated with higher levels of serum IL-17C, matrix metalloproteinase 10 and fibroblast growth factors than mild symptomatic COVID-19. Front. Immunol..

[16] Toro L (2023). High plasma levels of fibroblast growth factor 23 are associated with increased risk of COVID-19 in end-stage renal disease patients on hemodialysis: results of a prospective cohort. Toxins.

[17] Pan X (2018). FGF21 prevents angiotensin II-induced hypertension and vascular dysfunction by activation of ACE2/angiotensin-(1-7) axis in mice. Cell Metab..

[18] Song JJ (2021). Elabela prevents angiotensin II-induced apoptosis and inflammation in rat aortic adventitial fibroblasts via the activation of FGF21-ACE2 signaling. J. Mol. Histol..

[19] Edmonston D, Grabner A, Wolf M (2023). FGF23 and klotho at the intersection of kidney and cardiovascular disease. Nat. Rev. Cardiol..

[20] Pi M (2018). Cardiovascular interactions between fibroblast growth factor-23 and angiotensin II. Sci. Rep..

[21] Elghazi L, Cras-Méneur C, Czernichow P, Scharfmann R (2002). Role for FGFR2IIIb-mediated signals in controlling pancreatic endocrine progenitor cell proliferation. Proc. Natl Acad. Sci..

[22] Zhang T (2022). Risk for newly diagnosed diabetes after COVID-19: a systematic review and meta-analysis. BMC Med..

[23] Fignani D (2020). SARS-CoV-2 receptor angiotensin I-converting enzyme type 2 (ACE2) is expressed in human pancreatic β-cells and in the human pancreas microvasculature. Front. Endocrinol..

[24] Szlachcic WJ (2022). SARS-CoV-2 infects an in vitro model of the human developing pancreas through endocytosis. iScience.

[25] Wu C-T (2021). SARS-CoV-2 infects human pancreatic β cells and elicits β cell impairment. Cell Metab..

[26] Ibrahim S, Monaco GSF, Sims EK (2021). Not so sweet and simple: impacts of SARS-CoV-2 on the β cell. Islets.

[27] Fang L, Karakiulakis G, Roth M (2020). Are patients with hypertension and diabetes mellitus at increased risk for COVID-19 infection?. Lancet Respir. Med..

[28] Mancia G, Rea F, Ludergnani M, Apolone G, Corrao G (2020). Renin-angiotensin-aldosterone system blockers and the risk of Covid-19. N. Engl. J. Med..

[29] Reynolds HR (2020). Renin-angiotensin-aldosterone system inhibitors and risk of Covid-19. N. Engl. J. Med..

[30] Härdtner C, Mörke C, Walther R, Wolke C, Lendeckel U (2013). High glucose activates the alternative ACE2/Ang-(1-7)/Mas and APN/Ang IV/IRAP RAS axes in pancreatic β-cells. Int. J. Mol. Med..

[31] Shi TT (2018). Angiotensin-converting enzyme 2 regulates mitochondrial function in pancreatic β-cells. Biochem. Biophys. Res. Commun..

[32] Bindom SM, Hans CP, Xia H, Boulares AH, Lazartigues E (2010). Angiotensin I–converting enzyme type 2 (ACE2) gene therapy improves glycemic control in diabetic mice. Diabetes.

[33] Millman JR (2016). Generation of stem cell-derived β-cells from patients with type 1 diabetes. Nat. Commun..

[34] Rezania A (2014). Reversal of diabetes with insulin-producing cells derived in vitro from human pluripotent stem cells. Nat. Biotechnol..

[35] Sharon N (2019). Wnt signaling separates the progenitor and endocrine compartments during pancreas development. Cell Rep..

[36] Weng C (2020). Single-cell lineage analysis reveals extensive multimodal transcriptional control during directed beta-cell differentiation. Nat. Metab..

[37] Cao J (2020). A human cell atlas of fetal gene expression. Science.

[38] Kim S (2020). Molecular and genetic regulation of pig pancreatic islet cell development. Development.

[39] Blodgett DM (2015). Novel observations from next-generation RNA sequencing of highly purified human adult and fetal islet cell subsets. Diabetes.

[40] Yang L (2020). A human pluripotent stem cell-based platform to study SARS-CoV-2 tropism and model virus infection in human cells and organoids. Cell Stem Cell.

[41] Augstein P (2015). Localization of dipeptidyl peptidase-4 (CD26) to human pancreatic ducts and islet alpha cells. Diabetes Res. Clin. Pr..

[42] Veres A (2019). Charting cellular identity during human in vitro β-cell differentiation. Nature.

[43] Zhang X (2006). Receptor specificity of the fibroblast growth factor family: the Complete Mammalian FGF Family*. J. Biol. Chem..

[44] Wicik Z (2020). ACE2 interaction networks in COVID-19: a physiological framework for prediction of outcome in patients with cardiovascular risk factors. J. Clin. Med..

[45] Memon B, Abdelalim EM (2021). ACE2 function in the pancreatic islet: implications for relationship between SARS-CoV-2 and diabetes. Acta Physiol..

[46] Li Z (2022). Imatinib and methazolamide ameliorate COVID-19-induced metabolic complications via elevating ACE2 enzymatic activity and inhibiting viral entry. Cell Metab..

[47] Müller JA (2021). SARS-CoV-2 infects and replicates in cells of the human endocrine and exocrine pancreas. Nat. Metab..

[48] Chen J (2021). Inhibition of SARS-CoV-2 pseudovirus invasion by ACE2 protecting and Spike neutralizing peptides: an alternative approach to COVID19 prevention and therapy. Int. J. Biol. Sci..

[49] Shang J (2020). Cell entry mechanisms of SARS-CoV-2. Proc. Natl Acad. Sci..

[50] WHO. Living guidance for clinical management of COVID-19. (WHO, Geneva, Switzerland, 2021)

[51] Inchovska M, Ogneva V, Martinova Y (2006). Role of FGF1, FGF2 and FGF7 in the development of the pancreas from control and streptozotocin-treated hamsters. Cell Prolif..

[52] Alwahsh SM (2021). Fibroblast growth factor 7 releasing particles enhance islet engraftment and improve metabolic control following islet transplantation in mice with diabetes. Am. J. Transplant..

[53] Li Z, Wang S, Zhou F (2023). FXR inhibition: an innovative prophylactic strategy against SARS-CoV-2 infection. Signal Transduct. Target Ther..

[54] Brevini T (2023). FXR inhibition may protect from SARS-CoV-2 infection by reducing ACE2. Nature.

[55] Ni W (2020). Role of angiotensin-converting enzyme 2 (ACE2) in COVID-19. Crit. Care.

[56] Malhotra A, Hepokoski M, McCowen KC, John YJS (2020). ACE2, Metformin, and COVID-19. iScience.

[57] Nies VJM (2016). Fibroblast growth factor signaling in metabolic regulation. Front. Endocrinol..

[58] Wente W (2006). Fibroblast growth factor-21 improves pancreatic β-cell function and survival by activation of extracellular signal–regulated kinase 1/2 and Akt signaling pathways. Diabetes.

[59] Pagliuca FW (2014). Generation of functional human pancreatic β cells in vitro. Cell.

[60] Tran R, Moraes C, Hoesli CA (2020). Controlled clustering enhances PDX1 and NKX6.1 expression in pancreatic endoderm cells derived from pluripotent stem cells. Sci. Rep..

[61] Velazco-Cruz L (2019). Acquisition of dynamic function in human stem cell-derived β cells. Stem Cell Rep..

[62] Corman V, Bleicker T, Brunink S, Drosten C (2020). Diagnostic detection of 2019-nCoV by real-time RT-PCR. Eur. Surveill..

