# Supplementary material for: FGF7 enhances the expression of ACE2 in human islet organoids aggravating SARS-CoV-2 infection
Source: Signal Transduct Target Ther. 2024 Apr 23;9:104. doi: 10.1038/s41392-024-01790-8 (PMC11039711; doi:10.1038/s41392-024-01790-8)
Supplement: Supplementary file 1 — Supplementary Materials [file 41392_2024_1790_MOESM1_ESM.docx]

Supplementary Materials for

FGF7 enhances the expression of ACE2 in human islet organoids aggravating SARS-CoV-2 infection

Hao Meng^1,2^^#^, Zhiying Liao^1,2#^, Yanting Ji^2#^, Dong Wang^5#^, Yang Han^4^, Chaolin Huang^4^, Xujuan Hu^4^, Jingyi Chen^2,3^, Hengrui Zhang^2^, Zonghong Li^2^, Changliang Wang^2^, Hui Sun^2^, Jiaqi Sun^2^, Lihua Chen^2^, Jiaxiang Yin^2^, Jincun Zhao^2,5*^, Tao Xu^1,2*^, Huisheng Liu^1,2,3*^

Correspondence to: [liu_huisheng@gzlab.ac.cn](mailto:liu_huisheng@gzlab.ac.cn); [xu_tao@gzlab.ac.cn](mailto:xu_tao@gzlab.ac.cn); zhaojincun@gird.cn

**This PDF file includes:**

Materials and Methods

Supplementary Text

Supplementary Figures 1 to 5

Supplementary Tables 1 to 7

Materials and Methods

hESC maintenance and differentiation of hESC into pancreatic islet organoid

Basal medium formulations were prepared using MCDB131 with varying concentrations of glucose, sodium bicarbonate, BSA, ITS-X, and ascorbic acid as detailed in Supplementary Table 1. The differentiation procedure and administration of small molecules are outlined below. Approximately 80% density of H1 ES cells were dissociated into single-cell suspensions using TrypLE Express Enzyme (1×) (Life, Cat#12605028) and reseeded at a density of 2.5×105 cells/mL on Matrigel (Corning, Cat#354277) coated 24-wells plate supplemented in mTesR1 with Y-27632 for 24 hours. Once the cell density reached 70-90 %, islet organoid differentitation was initiated through stepwise administration of growth factors as described below.

Stage1 (S1, 3 days): Day1 S1 medium + 100 ng/mL Activin A (StemCell, Cat#78001) + 3 μM Chir99021 (Stemgent, Cat#04-0004-10) + Y-27632; Day2 S1 medium + 100 ng/mL Activin A + 0.1 μM Chir99021; Day3 S1 medium + 100 ng/mL activin A. Stage2 (S2, 2 days): S2 medium+50 ng/mL FGF7 (StemCell, Cat#78046). Stage3 (S3, 2 days): S3 medium+50 ng/mL FGF7 + 2 μM retinoic acid (Sigma, Cat#R2625) + 0.25 μM SANT-1 (Sigma, Cat#S4572) + 100 nM LDN193189 (Reprocell, Cat#40074) + 500 nM PdBU (Millipore Sigma, Cat#524390) + 10 μM Y27632. Stage4 (S4, 3 days): S4 medium+2 ng/mL FGF7 + 0.1 μM retinoic acid + 0.25 μM SANT-1 + 100 nM LDN193189 + 100 nM PdBU + 10 μM Y27632 + 10 μM ALK5i-II (Enzo Life Sciences, Cat#ALX-270-445-M005). Stage5 (S5, 3 days): S5 medium + 0.05 μM retinoic acid + 0.25 μM SANT-1 + 100 nM LDN193189 + 10 μM zinc sulfate (Sigma, Cat#Z0251) + 10 μM ALK5i-II + 1 μM T3 (Biosciences, Cat#64245) + 1 μM XXI (Millipore Sigma, Cat#595790) + 20 ng/mL Betacellulin (MCE, Cat#HY-P7005) + 10 μg/mL heparin（Sigma, Cat#H3149-500KU）. Stage6 (S6, 5 days): S6 medium + 100 nM LDN193189 + 1 μM T3 + 0.1 μM XXI + 10 μg/mL heparin + 2 μM R428 (SelleckChem, Cat#S2841) + 1 mM N-acetyl cysteine (Sigma, Cat#A9165) + 10 μM zinc sulfate. Stage7 (S7, 7-14 days): S7 medium + 1 μM T3 + 10 μg/mL heparin + 1 mM N-acetyl cysteine + 10 μM zinc sulfate + 10 μM (±)-α-Tocopherol (Sigma, Cat#T3251).

Magnetic enrichment of α and β cells

We employed the CD49a PE-conjugated antibody (1:25, BD Pharmingen, Cat#559596) and the anti-human CD26 antibody (Miltenyi, Cat#130-126-362) for the purification of β cells and α cells, respectively. Firstly, the β cells were stained with CD49a PE-conjugated antibody (1:25) and isolated using anti-PE MicroBeads UltraPure (Miltenyi, Cat#130–105-639) at a concentration of 40 μl per 10 million cells. The microbeads labelled β cells were then separated magnetically using MS Separation columns (Miltenyi, Cat#130-042-201) in a MiniMACS Separator (Miltenyi, Cat#130-042-102) following the manufacturer’s protocols, resulting in the collection of purified β cells. Next, the remaining cells underwent centrifugation and were stained with 1:50 dilution of the anti-human CD26 antibody (Miltenyi, Cat#130-126-362) to enable the magnetic isolation of α cells. The sorting procedure was repeated and purified α cells were collected. Additionally, the remained cells depleted of α and β cells, denoted as α-β- cells, were also collected for subsequent experiments.

SARS-CoV-2 pseudovirus production

For the infection experiment, purified α, purified β and α-β- cells (at a concentration of 5x105 cells/mL) were mixed with SARS-CoV-2 pseudovirus (diluted at a ratio of 1:10 with cell suspension) and then seeded on matrigel-coated glass coverslip (12 diameters, Cellvis, Cat#51210) for 2 hours. The cells should adhere on the coverslip within 2 hours. Then the cells were washed with DPBS and replenished with S7 medium. After 72 hours, the coverslips were collected for further analysis. Cells subjected to the same procedure but without pseudovirus infection were utilized as control samples.

qPCR analysis

Total RNA was extracted using the RNeasy Plus Mini Kit (Qiagen, Cat#74106). Subsequently, 1 μg RNA was reversed transcription using the Maxima H Minus (Thermo, Cat#EP0753). The resulting cDNA was used as a template in quantitative polymerase chain reaction (qPCR) assays. The TB Green premix EX Taq Mix (TAKARA, Cat#RR820B) was used in an CFX96 Real-Time Detection instrument (BIO-RAD, USA) following the manufacturer protocol. Primers utilized in the qPCR were synthesized by Sangon Biotech Co., Ltd. (Guangzhou, China) (Supplementary Table 2). Each sample was assayed in triplicate wells for qPCR analysis and the nuclear GAPDH gene served as the internal control. Data analysis was conducted using Graphpad Prism Software 8 and normalization was performed against undifferentiated H1 cells using ΔΔCt or relative to GAPDH using ΔCt.

Immunofluorescence staining

On the day of staining, the samples were permeabilized using 0.5% Tween and subsequently blocked with 10% appropriate serum. The primary antibodies were incubated overnight at 4 °C, followed by incubation with secondary antibodies and Hoechst 33342 (Thermo Scientific, Cat#875756-97-1) for 1h at room temperature. Details of antibodies used are provided in Supplementary Table 3. Finally, stained sample were mounted using immunoselect antifading mounting medium (ACMEC, Cat#AS2100). Confocal imaging was conducted with a Carl Zeiss LSM 800 or 880 inverted confocal laser scanning microscope and Zen 2.6 software was used to generate three- dimensional Z-stack images. Image processing and analysis were performed using ImageJ software.

Human intestinal organoid differentiation

Human intestinal organoids differentiation from hESC referenced previous publications with simple modifications 1,2. Briefly, hESCs were dissociated into single cell and replated at a density of 3.5×105 cells/well which reach approximately 80%-90% confluency within 24 hours. The differentiation of HIOs involved sequential stages including DE formation, mid/hind gut spheroids development and subsequent HIO maturation, as outlined below. When cells density reach 70-90 %, intestinal organoids were then differentiated by stepwise administration of growth factors in stage specific basal media (Supplementary Table 5). The mid/hindgut spheroids were collected and dissociated into single cells by accutase and encapsulated in Matrigel droplets (3000-5000 cells/25 μL droplet). The matrigel with cells were cultured in HIO maturation media for up to 21 days before being released from the matrigel Matrix. The HIOs were suspended and maintained in the maturation media until utilized for experimental purpose. The efficiency of HIO differentiation was assessed and quality-controlled through QPCR analysis and IF staining.

Human lung organoid differentiation

The differentiation process of human lung organoids (HLOs) from hESC was adapted from established protocols outlined in prior publications3-5, with minor adjustment. Initially, hESCs were dissociated into single cell and seeded at a density of 3.5×105 cells/well, allowing them to attain approximately 80%-90% confluency within 24 hours. The differentiation of HLO involved sequential stages encompassing DE induction, anterior foregut endoderm (AFE) spheroid formation, lung progenitor cells (LPC) generation and human lung organoids (HLO) maturation, as described below. Upon achieving a cell density of 70-90%, the differentiation of HLOs commenced through a stage application of growth factors, as detailed in Supplementary Table 6. The AFE spheroids were collected and encapsulated in Matrigel droplets (100-150 spheroids /25 μL droplet). Subsequently, LPC and HLO medium were subsequentially supplemented until mature airway cells were identified in the HLOs, which was confirmed by QPCR analysis and IF staining.

HUVEC, human lung fibroblasts and chondrocytes maintenance

HUVEC (MZ-0746) and human chondrocytes (MZ-0450) were procured from Mingzhoubio and cultured according to the manufacturer’s instructions. The HFL1 cell line (American Type Culture Collection CCL 153) is a fibroblast cell line isolated from fetal lung and was cultured following the HFL1 product sheet provided by ATCC.


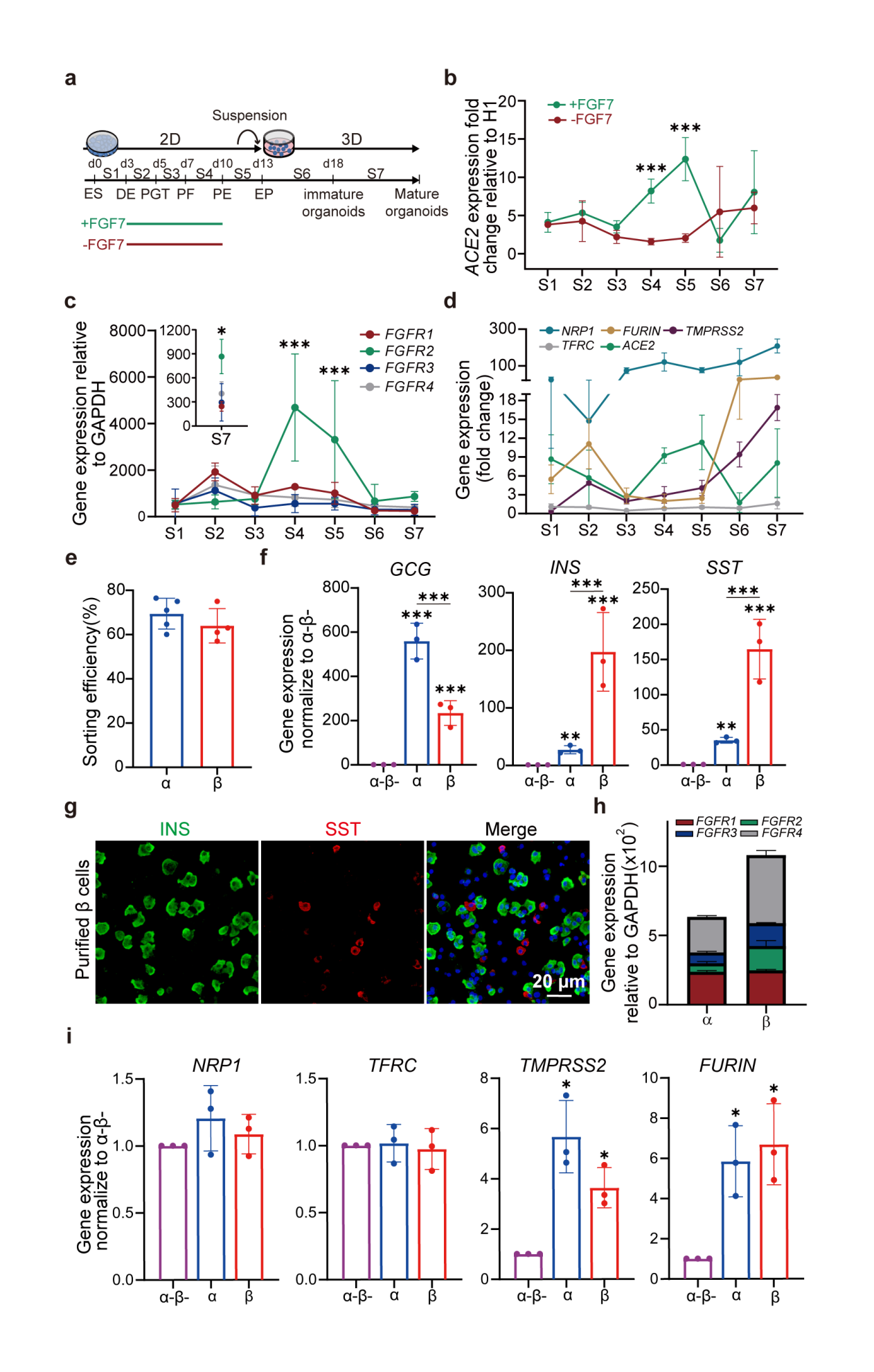


Supplementary Figure 1.

Major SARS-CoV-2 infection related receptors / proteases, FGFRs expressed from S1 to S7.

a) Schematic diagram outlining the differentiation protocol. The green line represents normal differentiation of islet organoid with FGF7 (+FGF7) , the red line represents differentiation protocol without FGF7 (-FGF7).

b) Dynamic gene expression of ACE2 relative to ES from S1 to S7 with or without FGF7 treatment.

c) Dynamic gene expression of FGFR1, FGFR2, FGFR3 and FGFR4 relative to GAPDH from S1 to S7.

d) Dynamic gene expression of NRP1, FURIN, TMPRSS2, TFRC, and ACE2 relative to GAPDH from S1 to S7.

e) Quantification of IF staining of positively stained GCG+ cells in purified α and INS+ cells in purified β cells relative to nuclei.

f) Gene expression of GCG, INS and SST in purified α, β and remaining α-β-, fold change relative to GAPDH was normalized to α-β-.

g) IF staining for INS and SST in purified β cells indicated no polyhormonal cells coexist in the same cell.

h) Gene expression of FGFR1, FGFR2, FGFR3 and FGFR4 relative to GAPDH in purified α cells and purified β cells.

i) Gene expression of SARS-CoV-2 receptors NRP1, TFRC, TMPRSS2 and FURIN in purified α, β and remaining α-β- cells. Values are normalized to α-β- cells.

Images are one representative experiment from 3 to 5 independent differentiations. For all statistical plots, the data are presented as mean ± SD, the point are represented as the individual values of 3 to 5 replicates with 3 repeats in each experiment. P values were calculated by one-way ANOVA and Tukey’s multiple comparison test. **P*<0.05, ***P*<0.01, ****P*<0.001.


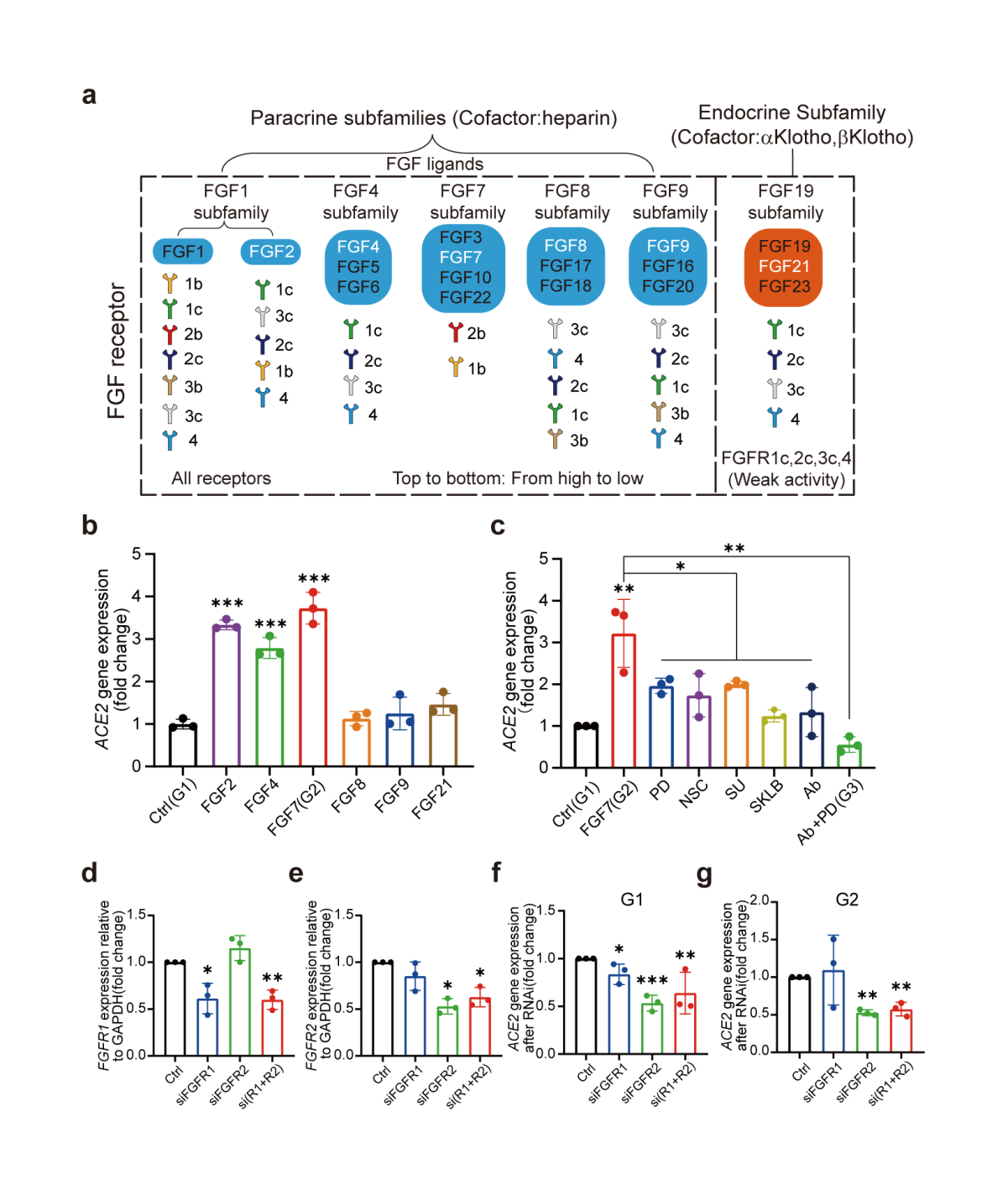


Supplementary Figure 2.

FGF subfamilies and FGFR1-4 modulate ACE2 expression primarily through FGFR2 downstream signals.

a) Schematic representation of human FGF subfamilies and their receptors.

b) ACE2 mRNA expression in pancreatic cells under the treatment of FGFs from different subfamilies.

c) Gene expression of ACE2 under FGFR1 inhibitors (PD, NSC and SU) and FGFR2 inhibitors (SKLB and Ab) treatment then stimulated by FGF7.

d-e) FGFR1 (d) and FGFR2 (e) gene expression in pancreatic EPs with the knocking down (KD) of FGFR1 or FGFR2 or both.

f-g) ACE2 gene expression in endocrine progenitors of G1 (f) and G2 (g) after KD of either FGFR1 or FGFR2 or both.

For all statistical plots, the data are presented as mean ± SD, the point are represented as the individual values of 3 replicates with 3 repeats in each experiment. P values were calculated by one-way ANOVA and Tukey’s multiple comparison test. **P*<0.05, ***P*<0.01, ****P*<0.001.


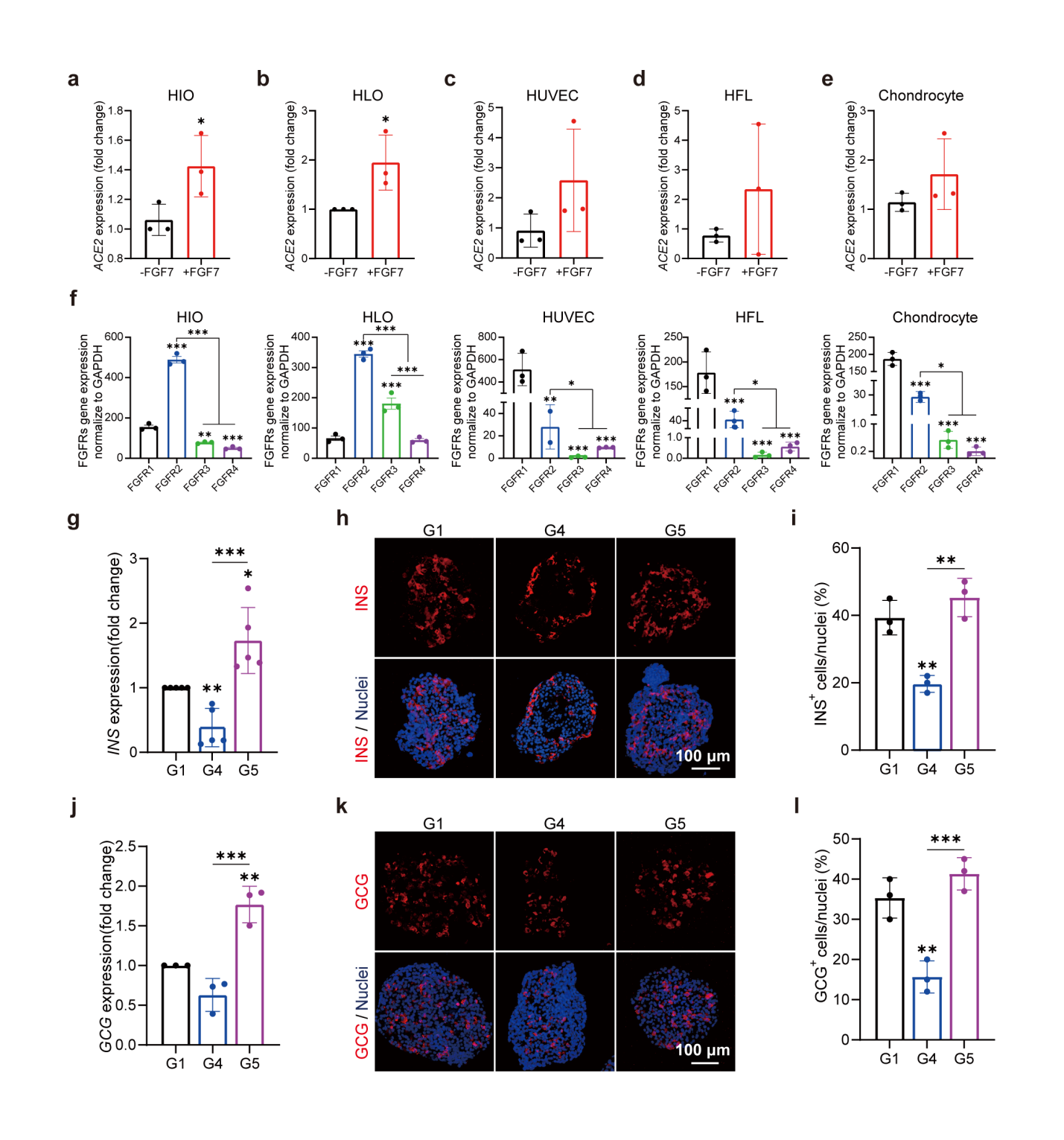


Supplementary Figure 3.

FGF7 regulates ACE2 expression in the organoids and cells with FGFR2 as the predominant receptor and reduces hormonal cells expression.

a-e) The impact of FGF7 on ACE2 mRNA expression in human intestinal organoids (HIO), human lung organoids (HLO), human umbilical vein endothelial cells (HUVEC), human lung fibroblast (HFL) and chondrocytes.

f) FGFR1 - 4 mRNA expression in HIO, HLO, HUVEC, HFL and chondrocyte.

g) Gene expression of INS in G1, G4 and G5. Values are normalized to G1.

h) IF staining of INS (red) and nuclei (blue) of islet organoids from G1, G4 and G5.
i) Quantification of (h) revealing the percentage of INS+ cells relative to the numbers of total cells counted by nuclei.

j) Gene expression of GCG in G1, G4 and G5. Values are normalized to G1.

k) IF staining of GCG (red) and DAPI (blue) of islet organoids from G1, G4 and G5.

l) Quantification of (k) revealing the percentage of GCG+ cells relative to the numbers of total cells counted by nuclei.

Images are one representative experiment from 3 to 4 independent differentiations. For all statistical plots, the data are presented as mean ± SD, the point are represented as the individual values of 3 to 5 replicates with 3 repeats in each experiment. P values were calculated by one-way ANOVA and Tukey’s multiple comparison test. **P*<0.05, ***P*<0.01, ****P*<0.001.


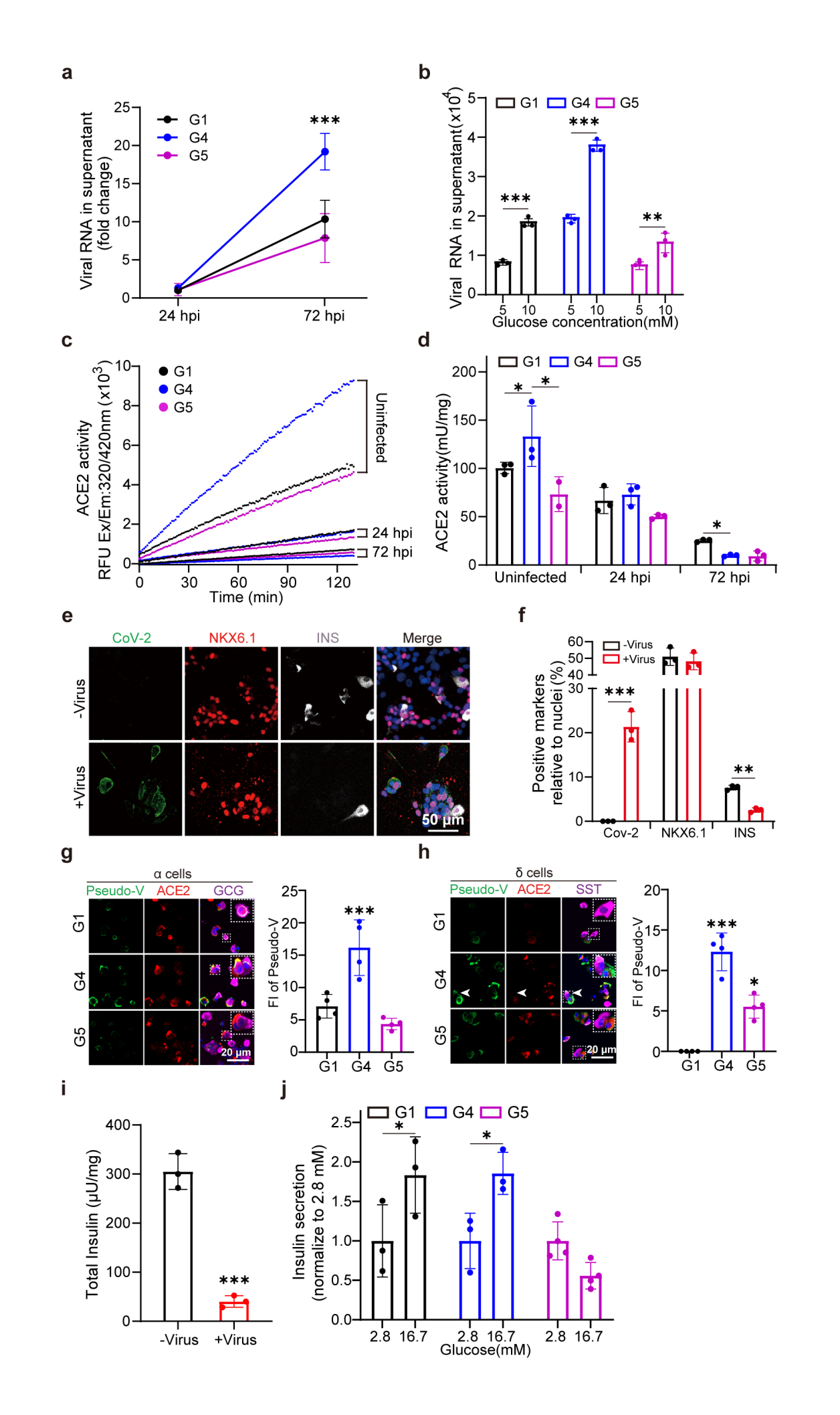


Supplementary Figure 4.

hESC-derived islet organoids respond to SARS-CoV-2 infection is time dependent.

a). qPCR analysis of SARS-CoV-2 viral RNA in the supernatants of islet organoids were harvested at 24 hpi and 72 hpi.

b) Higher concentration of glucose in culture medium combined FGF7 significantly promoted viral infection and replication in G4 compared to G1 and G5. FGFRi blocked FGF7 induced viral loading significantly but higher glucose increased the viral gene expression in G5.

c) The ACE2 enzymatic activity curves of G1, G4 and G5 from uninfected, 24hpi and 72hpi islet organoids in a kinetics model for 130 min.

d) ACE2 enzymatic activity (mU) per mg protein of uninfected, 24hpi and 72hpi islet organoids from G1, G4 and G5 groups.

e) The co-localization of SARS-CoV-2 (CoV-2, green), NKX6.1 (red) and INS (white) in virus infected and uninfected islet organoids.

f) Quantification of (e) for the positive markers ratio relative to nuclei before and after SARS-CoV-2 infection.

g) Tricolor IF costain of SARS-CoV-2 pseudo-virus spike (Pseudo-V, green), ACE2 (red) and GCG (purple) in purified α cells from G1, G4 and G5. The image in the top-right corner of the picture represents an enlarged view of the image enclosed within the dashed box. Quantification of images revealing fluorescent intensity of Pseudo-V in purified α cells after Pseudo-V penetration from G1, G4 and G5.

h) Tricolor IF costain of SARS-CoV-2 pseudo-virus spike (Pseudo-V, green), ACE2 (red) and SST (purple) in δ cells from G1, G4 and G5. The image in the top-right corner of the picture represents an enlarged view of the image enclosed within the dashed box. Quantification of images revealing fluorescent intensity of Pseudo-V in δ cells after Pseudo-V penetration from G1, G4 and G5.

i) Total insulin levels in islet organoids exposed to SARS-CoV-2 compare with uninfected islet organoids.

j) Insulin secretion of islet organoids at 24hpi in static GSIS assay. Images are one representative experiment from 3 to 4 independent biological replications. For all statistical plots, the data are presented as mean ± SD, the point are represented as the individual values of 3 to 4 replicates with 3 repeats in each experiment. P values were calculated by one-way ANOVA and Tukey’s multiple comparison test. **P*<0.05, ***P*<0.01, ****P*<0.001.


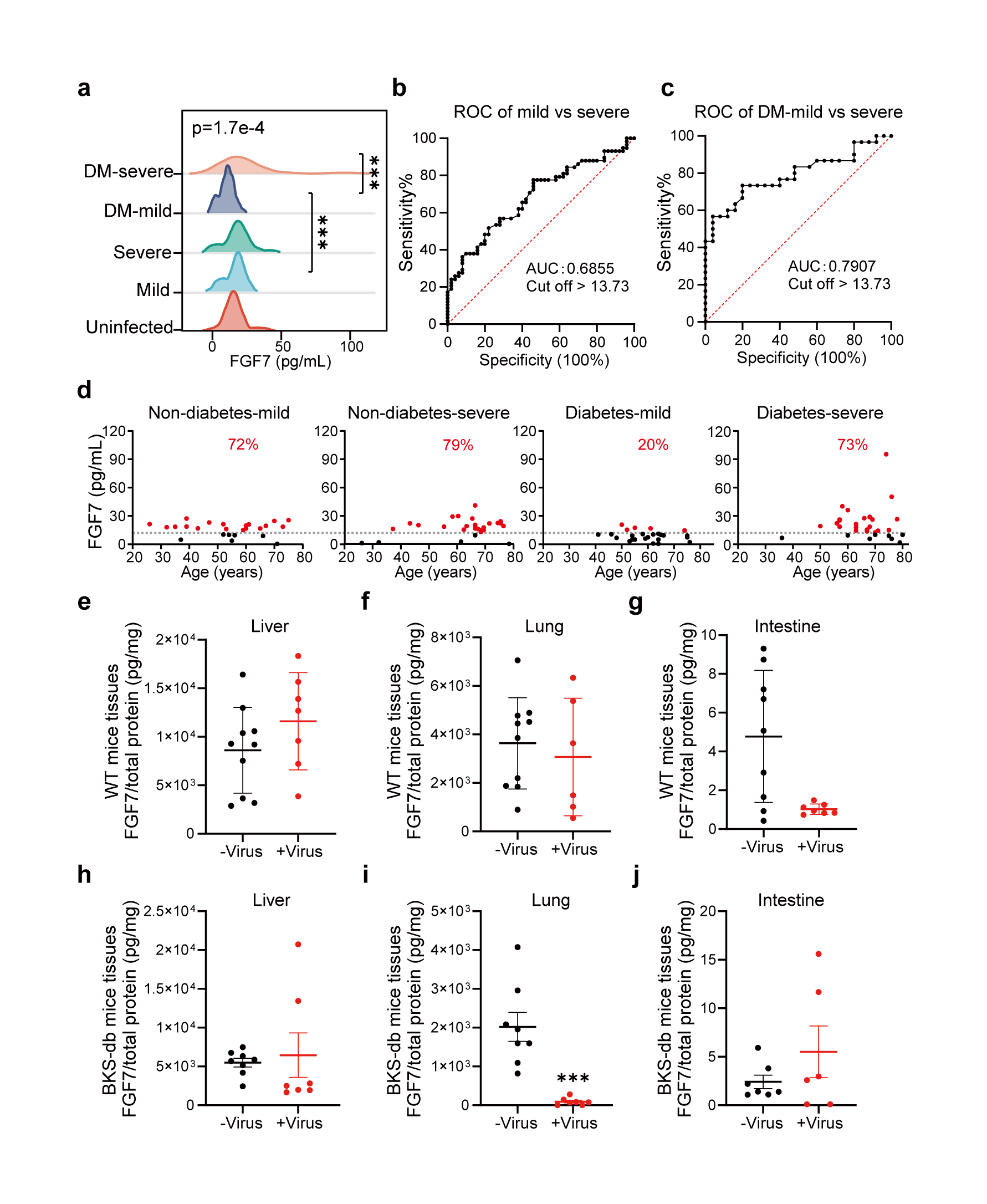


Supplementary Figure 5.

Plasma and tissue FGF7 concentration *in vivo.*

a) Ridge plot of FGF7 concentration in clinical plasma samples collected from COVID-19 patients with diabetes or non-diabetes.

b) Receiver operating characteristic curve (ROC) of COVID-19 patients with mild *vs* severe symptoms. AUC = 0.6855, Cut off > 13.37 pg/mL.

c) ROC of COVID-19 patients comorbid DM with mild *vs* severe symptoms. AUC = 0.7907, Cut off > 13.73 pg/mL.

d) Linear regression of FGF7 plasma concentration and age from non-diabetic mild, non-diabetic severe, diabetic mild and diabetic severe patients. The dash line represents the cut off value of 13.73 pg/mL. The red dots represent the patients with serum FGF7 higher than the cutoff value.

e-g) *In situ* FGF7 concentration in the major SARS-CoV-2 targeted organs (liver, lung and intestine) of WT mice.

h-j) *In situ* FGF7 concentration in the major SARS-CoV-2 targeted organs(liver, lung and intestine) of BKS-db mice.

Data are represented as the individual values of clinical samples or mice samples. ***p<0.001 with unpaired two-tailed t-test.

Supplementary Table 1.

Detailed basal medium formulation of hESC-derived islet organoids (in 500 mL MCDB 131 cell culture medium)

| stage  element | Glucose (Sigma, Cat#G8769) | sodium bicarbonate (Sigma, Cat#S6297) | bovine serum albumin (BSA) (Proliant, Cat#68700) | ITS-X (Invitrogen, Cat#51500056) | ascorbic acid (Sigma, Cat#A4544) |
| --- | --- | --- | --- | --- | --- |
| S1/S2 | 0.9 g | 0.75 g | 2.5 g | 10 μL | 0 mg |
| S3/S4 | 0.9 g | 1.25 g | 10 g | 5 mL | 22 mg |
| S5/S6 | 1.8 g | 0.875 g | 10 g | 5 mL | 22 mg |
| S7 | 1.8 g | 0.875 g | 10 g | 5 mL | 0 mg |

Supplementary Table 2.

FGFs family and FGFR inhibitor

| **FGFs family** | **Cat.No.** | **Company** | **Concentration** |
| --- | --- | --- | --- |
| FGF2 | HY-P7004 | MCE | 20ng/mL |
| FGF4 | 7460-F4-025 | R&D Systems | 500ng/mL |
| FGF4 | HY-P7014 | MCE | 500ng/mL |
| FGF7 | 78046 | Stem cell | 50ng/mL |
| FGF8 | HY-P7346 | MCE | 150ng/mL |
| FGF9 | HY-P7177 | MCE | 150ng/mL |
| FGF10 | 345-FG-250 | R&D Systems | 10ng/mL |
| FGF10 | HY-P7048 | MCE | 10ng/mL |
| FGF19 | HY-P7172 | MCE | 150ng/mL |
| FGF21 | HY-P7012 | MCE | 50ng/mL |
| FGF23 | HY-P7013 | MCE | 100ng/mL |
| **FGFRs inhibitor** | **Cat.No.** | **Company** | **Concentration** |
| SU5402 | S7667 | Selleckchem | 10mM |
| PD166866 | HY-101296 | MCE | 5mM |
| PD173074 | HY-10321 | MCE | 5mM |
| RPT835 | S8754 | Selleckchem | 5mM |
| NSC12 | S7940 | Selleckchem | 10mM |
| SKLB610 | S6526 | Selleckchem | 10mM |

Supplementary Table 3.

Primers used for qPCR

| **Gene** | **Forward** | **Reverse** |
| --- | --- | --- |
| *h-ACE2* | GAGATGGCAAGAGCAAATCATT | CATCAACTTTGCCCTCACATAG |
| *h-NRP1* | GGCAATGTGTTGAAGACCTTAG | TCATGCCTCCGAATAAGTACTC |
| *h-TFRC* | TGAACCAATACAGAGCAGACAT | GTTTTCTCAGCATTCCCGAAAT |
| *h-FURIN* | GACTTGGCAGGCAATTATGATC | CCTGTTGTCATTCATCTGTGTG |
| *h-TMPRSS2* | GCGTGGAAAAACCTCTTAACAA | TTTTTCTACTTGGTATCCGGCT |
| *h-GCG* | AGCTGCCTTGTACCAGCATT | GAGATTTCCCAGAAGAGGTCG |
| *h-INS* | AGGCCATCAAGCAGATCACT | GCAGCCTTTGTGAACCAACAC |
| *h-SST* | TGGGTTCAGACAGCAGCTC | CGCTGTCCATCGTCCTG |
| *h-KRT19* | ACGGCGAGCTAGAGGTGAAGATC | TGGTCGTGTAGTAGTGGCTGTAGTC |
| *h-GAPDH* | TGCACCACCAACTGCTTAGC | GGCATGGACTGTGGTCATGAG |
| *h-siFGFR1-1* | CCGACAAAGAGAUGGAGGUGCUUCATT | UGAAGCACCUCCAUCUCUUUGUCGGTT |
| *h-siFGFR1-2* | UAUACGUGCUUGGCGGUAACUCUATT | UAGAGUUACCGCCAAGCACGUAUATT |
| *h-siFGFR1-3* | GGUAGCAACGUGGAGUUCAUGUGUATT | UACACAUGAACUCCAUGUUGCUACCTT |
| *h-siFGFR2-1* | CCACGGACAAAGAGAUUGATT | UCAAUCUCUUUGUCCGUGGTT |
| *h-siFGFR2-2* | GAGUACUCCUAUGACAUUATT | UAAUGUCAUAGGAGUACUCTT |
| *h-siFGFR2-3* | GGUAGAAGACUUGGAUCGATT | UCGAUCCAAGUCUUCUACCTT |
| *h-FGFR1* | GCACATCCAGTGGCTAAAGCAC | AGCACCTCCATCTCTTTGTCGG |
| *h-FGFR2* | GTGCCGAATGAAGAACACGACC | GGCGTGTTGTTATCCTCACCAG |
| *h-FGFR3* | TCCATCTCCTGGCTGAAGAACG | TGTTCTCCACGACGCAGGTGTA |
| *h-FGFR4* | AACACCGTCAAGTTCCGCTGTC | CATCACGAGACTCCAGTGCTGA |
| *h-TGFβ* | CAGCAACAATTCCTGGCGATA | AAGGCGAAAGCCCTCAATTT |
| *h-SFTPB* | TGCCTGGACCACCTCATCCTTG | GTCCTCACACTCTTGGCATAGG |
| *h-SFTPC* | AGCAAAGAGGTCCTGATGGA | CGATAAGAAGGCGTTTCAGG |
| *h-CDX2* | ACACCTTCTACAATGAGC | ACGTCACACTTCATGATG |
| *h-VILLIN* | TAGCTGTGGTTGTAAAGCAGTACC | GGTATCATCTTTCTGAAGGAATAGG |
| *ms-GAPDH* | CATCACTGCCACCCAGAAGACTG | ATGCCAGTGAGCTTCCCGTTCAG |
| *ms-ACE2* | CACTCTGGGAATGAGGACACGG | TTTCCCCGTGCGCCAAGAT |
| *ms-TMPRSS2* | AAGTCCTCAGGAGCACTGTGCA | CAGAACCTCCAAAGCAAGACAGC |
| *ms-FGF7* | TGTTCTGTCGCACCCAGTGGTA | TTCCAACTGCCACGGTCCTGAT |
| *ms-FGFR2* | GTCTCCGAGTATGAGTTGCCAG | CCACTGCTTCAGCCATGACTAC |
| *ms-GCG* | GCACATTCACCAGCGACTAC | TTCCGGTTCCTCTTGGTGTT |
| *ms-INS* | CAAACCCACCCAGGCTTTTG | AACGCCAAGGTCTGAAGGTC |

Supplementary Table 4.

Antibodies

| **Primary antibodies** | | | | | |
| --- | --- | --- | --- | --- | --- |
| **Antigen** | **Species** | | **Cat.No.** | **Company** | **Dilution** |
| ACE2 | | rabbit | ab15348 | Abcam | 1:500 (IF)  1:2,000 (WB) |
| ACE2 | | goat | AF933 | R&D systems | 1:200 |
| Insulin | | rabbit | 3014S | Cell Signaling Technology | 1:500 (IF) |
| Insulin | | mouse | Ab181547 | Abcam | 1:500 (IF)  1:2,000 (WB) |
| Glucagon | | mouse | Ab10988 | Abcam | 1:500 (IF) |
| Glucagon | | mouse | A14609 | ABclonal | 1:2000 (WB) |
| Somatostatin | | rat | MAB62971 | Millipore | 1:300 (IF) |
| Cytokeratin 19  (CK19) | | mouse | Ab9221 | Abcam | 1:250 (IF) |
| β-actin | | mouse | 66009-1-lg | proteintech | 1:2,000(WB) |
| OCT4 | | rabbit | Ab19857 | Abcam | 1:500 (IF) |
| SOX17 | | mouse | Ab84990 | Abcam | 1:200 (IF) |
| FOXA2 | | rabbit | WRAB-FOXA2 | Seven Hills Bioreagents | 1:500 (IF) |
| HNF1B | | mouse | Ab236759 | Abcam | 1:500 (IF) |
| PDX1 | | goat | AF2419 | R&D | 1:200 (IF) |
| PDX1 | | rabbit | ab134150 | Abcam | 1:200 (IF) |
| NKX6.1 | | ms | ab268088 | Abcam | 1:200 (IF) |
| MAFA | | rabbit | Ab26405 | Abcam | 1:250 (IF) |
| C-peptide | | rat | GN-ID4-s | DSHB | 1:100 (IF) |
| GFP | | goat | Ab6673 | Abcam | 1:250 (IF) |
| GFP | | mouse | AE012 | Abclonal | 1:250 (IF) |
| SARS-CoV / SARS-CoV-2 Nucleocapsid Antibody, | | rabbit | 40143-T62 | SinoBiological | 1:200（IF） |
| SFTPB | | mouse | sc-133143 | Santa Cruz Biotechnology | 1:200（IF） |
| SFTPC | | rabbit | WRAB-9337 | Seven Hills Bioreagents | 1:200（IF） |
| CDX | | rabbit | Ab76541 | Abcam | 1:500 (IF) |
| ZO-1 | | Mouse | 33-9100 | Invitrogen | 1:300 (IF) |
| **Secondary antibodies** | | | | | |
| **Antigen** | | | **Cat.No.** | **Company** | **Dilution** |
| Alexa Fluor 488  Donkey anti-Rabbit | | | A-21206 | Invitrogen | 1:500 (IF) |
| Alexa Fluor 488  Donkey anti-Goat | | | A-11055 | Invitrogen | 1:500 (IF) |
| Alexa Fluor 488  Donkey anti-Mouse | | | A-21202 | Invitrogen | 1:500 (IF) |
| Alexa Fluor 546  Donkey anti-Mouse | | | A-10016 | Invitrogen | 1:500 (IF) |
| Alexa Fluor 546  Donkey anti-Rabbit | | | A-10040 | Invitrogen | 1:500 (IF) |
| Alexa Fluor 647  Donkey anti-Mouse IgG | | | A-31571 | Invitrogen | 1:500 (IF) |
| Donkey anti-Rat IgG-647 | | | ab150155 | Abcam | 1:500 (IF) |
| Donkey anti-Goat IgG-647 | | | ab150135 | Abcam | 1:500 (IF) |
| HRP-conjugated Affinipure Goat Anti-Mouse IgG (H+L) | | | SA00001-1 | Proteintech | 1:5000 (WB) |
| HRP-conjugated Affinipure goat anti-rabbit lgG (H+L) | | | SA00001-2 | Proteintech | 1:5000 (WB) |

Supplementary Table 5.

Human intestinal organoids differentiation medium formulation

| Media | DE  Day 1 | DE  Day 2 | DE  Day 3 | Mid/Hindgut  Day 4-8 | HIO  Day 30-40 |
| --- | --- | --- | --- | --- | --- |
| Basel media | RPMI1640  BSA (0.2%)  GlutaMAX (1 ×)  Pen-Strep (1×) | | | RPMI 1640  B27(1 ×)  GlutaMAX (1×)  Pen-Strep (1×) | Advanced DMEM/F12 medium (50%)  R-spondin1 condition medium (50%)  GlutaMAX (1 ×), Pen-Strep (1×)  N2 (1 ×), B27 (1 ×), HEPES (15 mM) |
| Add on day of use | 100 ng/mL Activin A | | | 500 ng/mL FGF4  3 μM CHIR-99021 | 100 ng/mL Noggin  50 ng/mL EGF  500 nM A83-01  3 μM Chir-99021 |

Supplementary Table 6.

| Media | DE  Day 1 | DE  Day 2 | DE  Day 3 | AFE  Day 4-8 | LPC  Day 1-7 | HLO  Day 30 |
| --- | --- | --- | --- | --- | --- | --- |
| Basel media | MCDB131  Glucose (10 mM)  NaHCO_3_ (1.5 mg/mL)  BSA (5 mg/mL)  GlutaMAX (1 ×)  ITS-X (1 ×) | | | Advanced DMEM/F12  N-2(1×), B27(1×)  HEPES (10 mM)  GlutaMAX (1×)  Pen-Strep (1×) | | |
| Add on day of use | 100 ng/mL Activin A | | | 10 μM SB431542  200 ng/mL Noggin  1μM SAG,  500 ng/mL FGF4  2μM CHIR-99021 | 3μM Chir  10ng/mL FGF7  10ng/mL FGF10  DAPT 20 μM | 50nM DXMS  100nM 8-Br  100nM IBMX  3μM Chir  10μM SB431542 |
|  | 3μM Chir | 3μM Chir |  |  |  |  |

Human lung organoids differentiation medium formulation

Supplementary Table 7.

Daily weighing of mice infected with the β variant (g)

| Wt/db | No.# | 1dpi | 2dpi | 3dpi | 4dpi | 5dpi |
| --- | --- | --- | --- | --- | --- | --- |
| wt | 1 | 16.5 | 16.7 | 15.0 | 15.5 | 16.1 |
|  | 2 | 19.3 | 20.0 | 18.0 | 19.2 | 19.6 |
|  | 3 | 18.4 | 19.5 | 17.3 | 18.2 | 18.5 |
|  | 4 | 18.5 | 19.3 | 17.2 | 18.3 | 18.8 |
|  | 5 | 18.7 | 19.2 | 17.5 | 18.9 | 19.1 |
|  | 6 | 19.0 | 19.5 | 17.5 | 17.2 | 18.0 |
|  | 7 | 18.5 | 19.3 | 17.3 | 17.9 | 18.1 |
|  | 8 | 18.8 | 19.1 | 17.0 | 18.9 | 18.4 |
|  | 9 | 18.3 | 18.9 | 16.8 | 17.1 | 17.5 |
| db | 10 | 19.2 | 18.6 | 16.8 | 16.4 | 16.2 |
|  | 11 | 38.1 | 38.1 | 38.8 | 39.1 | 39.0 |
|  | 12 | 36.6 | 35.6 | 36.2 | 37.2 | 37.8 |
|  | 13 | 39.4 | 37.2 | 37.6 | 38.9 | 38.3 |
|  | 14 | 37.4 | 36.4 | 36.9 | 37.7 | 39.0 |
|  | 15 | 37.5 | 37.0 | 36.7 | 37.8 | 38.3 |
|  | 16 | 38.8 | 36.7 | 37.9 | 39.0 | 39.5 |
|  | 17 | 36.7 | 36.0 | 36.8 | 37.4 | 37.0 |
